# Type-Specific Human Papillomavirus Biological Features: Validated Model-Based Estimates

**DOI:** 10.1371/journal.pone.0081171

**Published:** 2013-11-29

**Authors:** Iacopo Baussano, K. Miriam Elfström, Fulvio Lazzarato, Anna Gillio-Tos, Laura De Marco, Francesca Carozzi, Annarosa Del Mistro, Joakim Dillner, Silvia Franceschi, Guglielmo Ronco

**Affiliations:** 1 International Agency for Research on Cancer, Lyon, France; 2 Department of Medical Epidemiology & Biostatistics, Karolinska Institutet, Stockholm, Sweden; 3 Unit of Cancer Epidemiology, Department of Medical Sciences, University of Turin, Turin, Italy; 4 Institute for Cancer Study and Prevention (ISPO), Florence, Italy; 5 Molecular Oncological and Diagnostic Immunology, Venetian Oncology Institute (IOV), Padova, Italy; 6 Unit of Cancer Epidemiology, Centre for Cancer Prevention, Turin, Italy; Arizona State University, United States of America

## Abstract

Infection with high-risk (hr) human papillomavirus (HPV) is considered the necessary cause of cervical cancer. Vaccination against HPV16 and 18 types, which are responsible of about 75% of cervical cancer worldwide, is expected to have a major global impact on cervical cancer occurrence. Valid estimates of the parameters that regulate the natural history of hrHPV infections are crucial to draw reliable projections of the impact of vaccination. We devised a mathematical model to estimate the probability of infection transmission, the rate of clearance, and the patterns of immune response following the clearance of infection of 13 hrHPV types. To test the validity of our estimates, we fitted the same transmission model to two large independent datasets from Italy and Sweden and assessed finding consistency. The two populations, both unvaccinated, differed substantially by sexual behaviour, age distribution, and study setting (screening for cervical cancer or *Chlamydia trachomatis* infection). Estimated transmission probability of hrHPV types (80% for HPV16, 73%-82% for HPV18, and above 50% for most other types); clearance rates decreasing as a function of time since infection; and partial protection against re-infection with the same hrHPV type (approximately 20% for HPV16 and 50% for the other types) were similar in the two countries. The model could accurately predict the HPV16 prevalence observed in Italy among women who were not infected three years before. In conclusion, our models inform on biological parameters that cannot at the moment be measured directly from any empirical data but are essential to forecast the impact of HPV vaccination programmes.

## Introduction

In year 2008, new cancer cases attributable to high-risk (hr) human papillomavirus (HPV) infection were estimated to be 610,000 [1]. Some 87% (530,000) of them were cervical cancers. HrHPV infection is a necessary, though not sufficient, cause of cervical cancer [2,3]. Around 75% of all cervical cancers worldwide is attributable to HPV16 and 18 types [4]. The introduction of highly effective vaccines against HPV16 and 18 [5,6] is expected to have a major impact on cervical and the other HPV-related cancers on a global scale within the next decades [7]. 

Some key parameters that govern the natural history of hrHPV infection, including the probability of transmission per sexual partnership, the rate of clearance of incident infections and immune response following infection clearance, are, however, currently ill-defined because they cannot be easily inferred from empirical data. These parameters are needed, among other uses, for projecting the impact of cervical cancer control measures (vaccination and/or screening) by simulation with mathematical models, as done in some populations [8–10]. Transmission models have been parameterized either by imposing plausible sets of parameter values to the simulated population [11–14] or by calibration of model-based outputs against empirical sets of data [13,15–18]. 

Mathematical models have, however, also been used to estimate ill-defined parameters [15,16,19–23]. In order to avoid circular reasoning [10], model validation has been mainly performed by comparing the shape and peak magnitude of model’s projections of hrHPV and cervical cancer age-specific distribution to data reported in the literature [13,14,24–26] or data other than those used during the fitting procedure (cross-validation) [21].

The natural history of infection is expected to be relatively similar in different populations. Therefore, the biological parameters obtained by fitting the same model to datasets from different populations are expected to be consistent. In addition, the above-mentioned parameters determine the evolution of type-specific HPV prevalence. Therefore, the model should allow correct projections of subsequent prevalence in the same population. In particular, parameters estimated on the basis of prevalence at time *t*_0_ should provide correct predictions of the prevalence at time *t*_*i*_ among women from the same population uninfected at *t*_*0*_. 

In the present work, for the first time, we based validation on both cross-validation, i.e. assessment of the consistency of estimates obtained from different populations, and on out-of-sample validation, i.e. assessment of the consistency between model-based projections and independent sets of data from the same population not used to fit the model. We have separately estimated the above mentioned parameters for 13 hrHPV types by independently fitting the same dynamic transmission model to two populations recruited in two studies in Italy [27] and Sweden [28] and compared results. In addition, we have compared the HPV16 age-specific prevalence observed three years later among initially negative women in the Italian study with the model-based projection of the same curve. We used this validation only for HPV16 because the observation was done just on a sample of women (see Methods). Thus precision of age-specific prevalence observed three years later for the other types was limited. 

## Results

### Model

We developed a partial integro-differential equation model of heterosexually transmitted HPV infection. Each hrHPV type (i.e. 16, 18, 31, 33, 35, 39, 45, 51, 52, 56, 58, 59, and 68) was modelled independently from the other types. The model accounted for the effect of age and sexual behaviour, categorized in classes of sexual activity (CSA), time since infection (as a determinant of infection clearance), and different patterns of immune response to re-infection. 

We separately fitted the same model’s outputs to the hrHPV type and age- specific prevalence curves observed in Italy and Sweden. Data from Italy were obtained from one of the largest (94,370 women) population-based randomized controlled trials on New Technologies for Cervical Cancer screening (NTCC trial) [27], while data from Sweden were obtained from the national voluntary *Chlamydia trachomatis* screening (33,137 women) [28]. In order to allow for the uncertainty of model-based estimates, we selected the 100 best-fitting curves for each country and estimated the median and the inter-quartile range (IQR) of each parameter for each hrHPV type in the two countries. We tested the between-country consistency of our estimates for each HPV type by the non-parametric Mann-Whitney test. An overview of the model and fitting process is presented in the Materials and Methods section. The complete model description with a and the full set of equations and parameters are provided in File S1. 

### Characteristics of hrHPV natural history

The clearance rate of HPV16 and 18 in the first six months was 0.120 and 0.133 per month, respectively, in Sweden and 0.119 and 0.135 per month, respectively, in Italy and decreased to 0.039 and 0.043 and to 0.038 and 0.046 per month, respectively, in the two countries, three years after infection (Table 1). The rates of clearance of the other hrHPV types were also found to decrease (less than exponentially) over time since infection in both countries. Estimated medians and IQR of clearance rates at 0.5, 1.5 and 3 years after infection for each of the 13 considered hrHPV types are presented in Table 1, separately for Sweden and Italy. 

**Figure 1:**
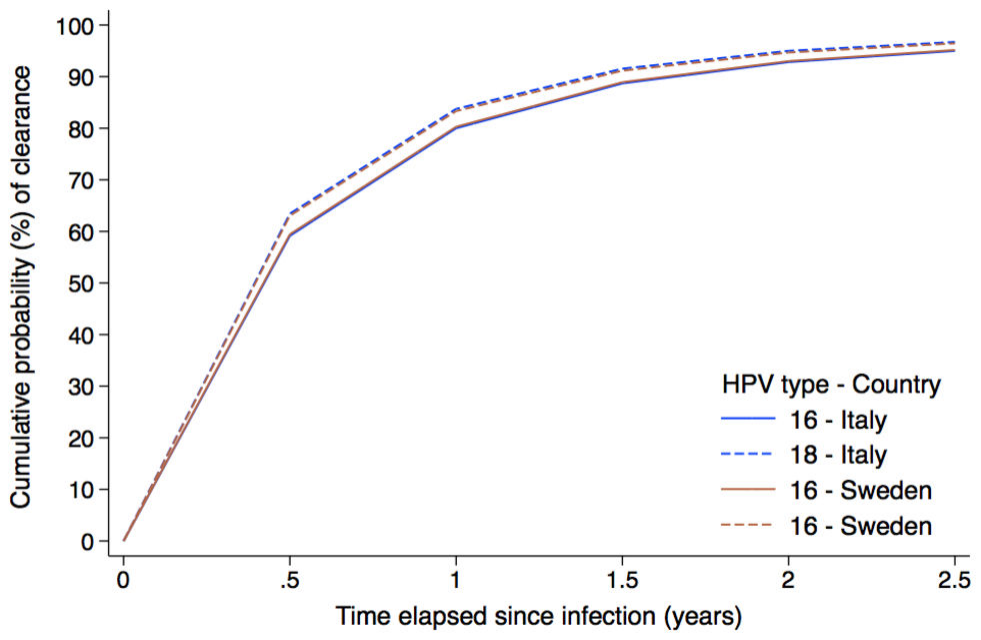
Estimated median cumulative probability (%) of clearance of HPV16 and 18 infections, by country. Abbreviation: HPV = human papillomavirus.

Types HPV16, 18, 39, and 51 were shown to persist longer than types 33, 35, and 68 (Table 1 and Figure S2.1 in File S4). Figure 1 shows the cumulative probability (%) of HPV16 and 18 clearance: within two years about 90% of all incident infections were cleared. Curves were virtually identical in the two countries. Our estimates of clearance rates were also not significantly different between countries for types 35, 39, 51 and 56 (Table 1) at α level 0.05 and for types 45, 52, 59, and 68 at α level 0.01.

**Table 1:** Estimated median (and inter-quartile range) rate of infection clearance (person-month), by HPV type, years elapsed since infection, and country.

	Years elapsed since infection (Sweden)			Years elapsed since infection (Italy)		
HPV type	0.5	1.5	3	0.5	1.5	3
HPV16*	0.120(0.096 - 0.148)	0.077(0.046 - 0.114)	0.039(0.015 - 0.077)	0.119(0.093 - 0.148)	0.076(0.044 - 0.112)	0.038(0.014 - 0.073)
HPV18*	0.133(0.098 - 0.167)	0.084(0.043 - 0.133)	0.043(0.013 - 0.095)	0.135(0.101 - 0.173)	0.088(0.049 - 0.133)	0.046(0.017 - 0.088)
HPV31	0.133(0.102 - 0.165)	0.087(0.049 - 0.130)	0.047(0.016 - 0.091)	0.119(0.094 - 0.146)	0.078(0.045 - 0.114)	0.043(0.015 - 0.080)
HPV33	0.166(0.117 - 0.203)	0.103(0.048 - 0.153)	0.051(0.013 - 0.101)	0.15(0.110 - 0.189)	0.094(0.049 - 0.144)	0.048(0.014 - 0.096)
HPV35*	0.17(0.105 - 0.223)	0.086(0.033 - 0.152)	0.031(0.006 - 0.085)	0.164(0.106 - 0.222)	0.095(0.037 - 0.165)	0.043(0.008 - 0.107)
HPV39*	0.149(0.107 - 0.196)	0.09(0.042 - 0.143)	0.042(0.010 - 0.089)	0.157(0.113 - 0.192)	0.095(0.049 - 0.141)	0.044(0.014 - 0.088)
HPV45**	0.149(0.101 - 0.202)	0.083(0.039 - 0.143)	0.034(0.009 - 0.084)	0.159(0.115 - 0.198)	0.097(0.050 - 0.139)	0.046(0.014 - 0.083)
HPV51*	0.125(0.094 - 0.159)	0.085(0.046 - 0.128)	0.048(0.016 - 0.093)	0.13(0.102 - 0.164)	0.092(0.056 - 0.131)	0.054(0.023 - 0.093)
HPV52**	0.135(0.098 - 0.173)	0.083(0.043 - 0.129)	0.04(0.013 - 0.083)	0.151(0.108 - 0.188)	0.09(0.046 - 0.138)	0.042(0.013 - 0.086)
HPV56*	0.155(0.104 - 0.199)	0.088(0.039 - 0.148)	0.038(0.009 - 0.096)	0.161(0.110 - 0.208)	0.086(0.042 - 0.140)	0.033(0.009 - 0.078)
HPV58**	0.16(0.111 - 0.200)	0.096(0.043 - 0.153)	0.044(0.011 - 0.102)	0.133(0.102 - 0.166)	0.084(0.048 - 0.126)	0.042(0.015 - 0.083)
HPV59**	0.16(0.105 - 0.199)	0.111(0.048 - 0.168)	0.063(0.014 - 0.129)	0.16(0.113 - 0.208)	0.097(0.048 - 0.154)	0.045(0.014 - 0.098)
HPV68**	0.167(0.105 - 0.218)	0.083(0.029 - 0.148)	0.028(0.004 - 0.083)	0.167(0.116 - 0.218)	0.096(0.047 - 0.158)	0.042(0.012 - 0.098)
Explored Ranges**^*¶*^**	0.028 - 0.292	0.006 - 0.292	0.000 - 0.292	0.028 - 0.292	0.006 - 0.292	0.000 - 0.292

***^¶^*** 100,000 different combinations of parameter values were drawn from the prior uniform distributions, using the Latin Hypercube sampling method within the explored range. The explored range of rate of infection clearance was based on previous work (4) and allowed to remain constant over time elapsed since infection. ^*^ ^/^ ^**^Estimates consistent between countries, as assessed through Mann-Whitney test (*α -level=0.05; ** α -level=0.01)

Abbreviation: HPV = human papillomavirus

**Figure 2:**
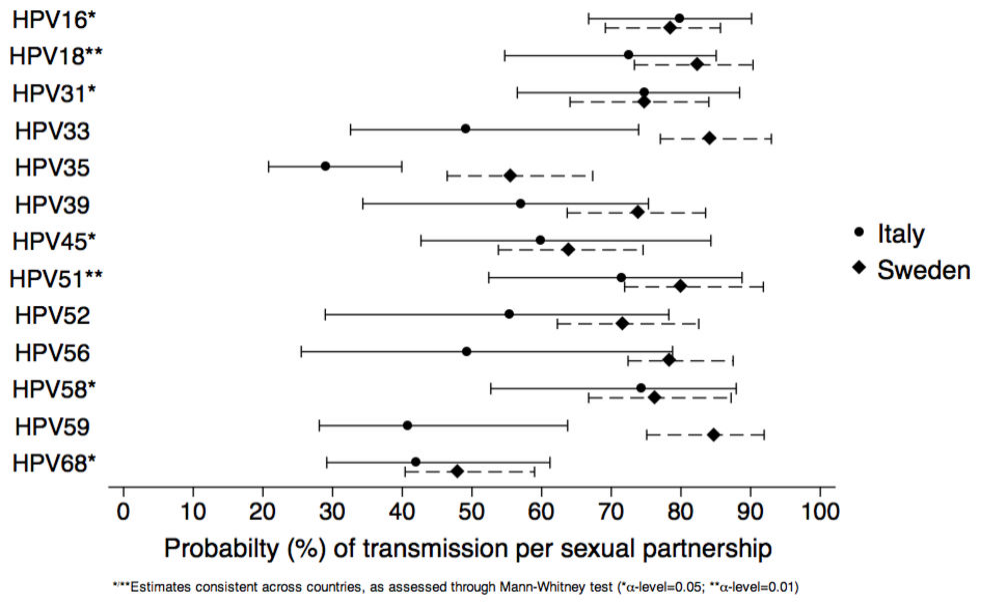
Estimated median (and inter-quartile range) probability (%) of HPV infection transmission, by HPV type and country. Abbreviation: HPV= human papillomavirus.

Figure 2 shows the probability (%) of HPV infection transmission per sexual partnership, by hrHPV type and country. It was approximately 80% for HPV16 and between 70% and 90% for HPV18, 31, 51, and 58. The similarity in the transmission probability between the two countries was higher for the most frequent hrHPV types, notably HPV16 and 31. Our estimates of transmission probability were also not significantly different for types 45, 58, and 68 at α level 0.05 and for types 18 and 51 at α level 0.01. For other less frequent hrHPV types (i.e., 33, 35, 39, 52, 56, and 59) significant differences of transmission probability ranged between 17 to 44%.

**Figure 3:**
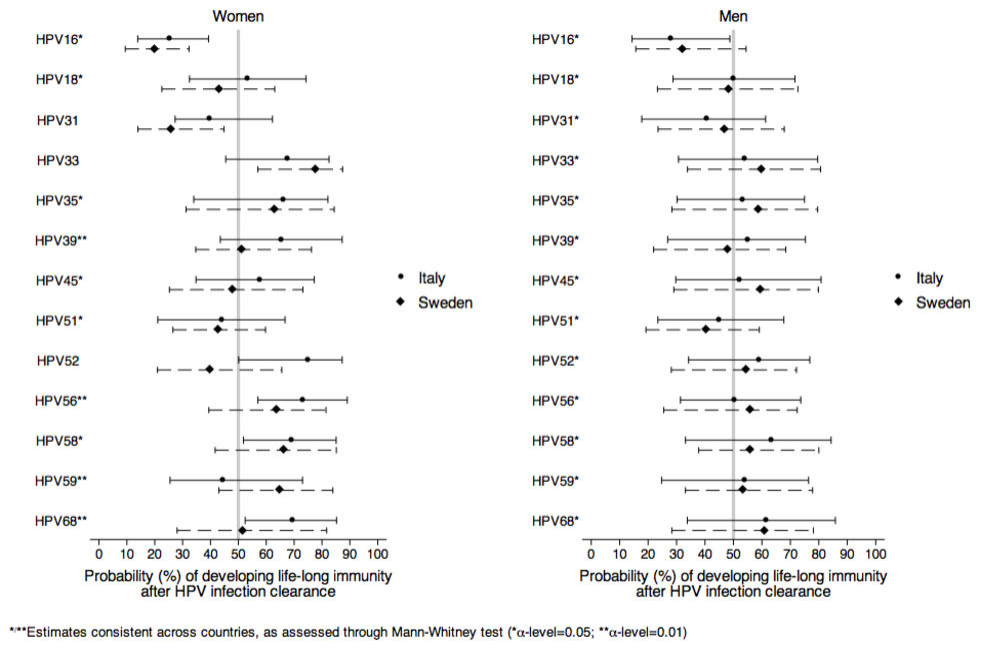
Estimated median (and inter-quartile range) probability (%) of developing life-long immunity after HPV infection clearance by HPV type. As the probability decreases from 50% to 0%, HPV infections are increasingly more likely to be cleared without developing immunity, i.e. increasingly characterized by a predominant SIS clearance pattern. By contrast as the probability increases from 50% to 100%, HPV infections are increasingly more likely to be cleared by developing immunity, i.e. increasingly characterized by predominant SIR clearance pattern. Abbreviation: HPV= human papillomavirus; SIS=susceptible-infected-susceptible; SIR=susceptible-infected-resistant.

To test the hypothesis of different immune responses by sex, different probabilities of developing protective immunity against individual hrHPV types after HPV infection clearance were allowed in men and women. Protective immunity was similar in the two countries (Figure 3). HPV16 infection was found to clear preferentially according to a susceptible-infected-suceptible (SIS) pattern in both genders. Other hrHPV types showed an approximately equal fraction of susceptible-infected-resistant (SIR) and SIS clearance patterns among men and a slightly larger fraction of SIR patterns among women (Figure 3). However, no clear difference in immune response between sexes was found. Type-specific estimates among men were always consistent between the two countries. The same estimates among women were consistent between countries for types 16, 18, 35, 45, 51, and 58 at α level 0.05 and for types 39, 56, 59, and 68 at α level 0.01. Additional information on characteristics of the natural history for each HPV type and by country is reported in Supplementary Materials (Tables S2.1, S2.2 & S2.3 in File S3 for Sweden and Italy).

**Figure 4:**
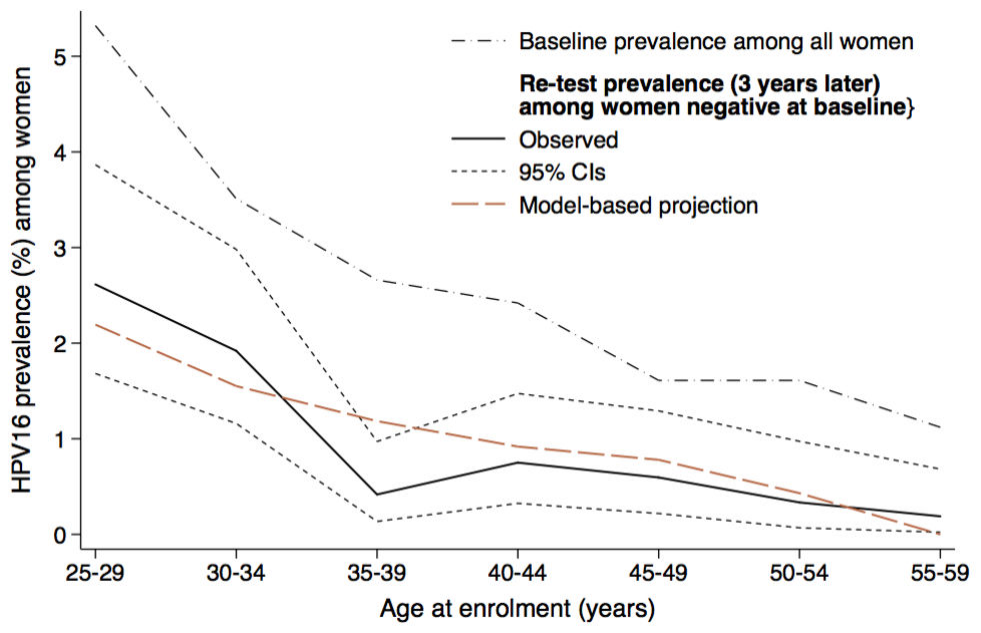
Age-specific HPV16 prevalence (%). Model-based projection and prevalence observed in NTCC trial at baseline and re-test 3 years later are shown. Abbreviation: HPV= human papillomavirus; NTCC = New Technologies for Cervical Cancer Screening.

### Sexual behaviour

Information on sexual behaviour was obtained from nation-wide population-based surveys [29,30] and applied to our study populations. We represented the network of sexual partnerships by making some simplifications (see Materials and Methods section for further details). We estimated sexual preferences in terms of age and sexual activity assortativeness (i.e. the tendency for individuals with similar age and sexual activity to form sexual partnerships) [31]. Mildly assortative patterns were found across age and CSA groups (ranging between 0.2 and 0.4, on a scale where fully and randomly assortative behaviours correspond to value 0 and 1) in both countries. Assortativeness by age and sexual activity was significantly higher in Italy than in Sweden. In Italy assortativeness by age (i.e. 0.2) was more important than by sexual activity (i.e. 0.3) (see Tables S2.1, S2.2 & S2.3 in File S3).

### Sensitivity analysis

The fit between type- and age- specific prevalence curves observed in Italy and Sweden and the 100 best fitting model outputs are shown in the Supplementary Materials (Figure S2.2-S2.4 in File S5, S2.5-S2.8 in File S6). Univariate and multivariable sensitivity analyses based on Latin hypercube sampling were used to assess the influence of model parameters on our estimates. We calculated a sensitivity index to quantify the relative importance of each input parameter in the fit of our transmission model predictions to observed data (Table 2). For HPV16 and 18 in both countries, the model’s fit was more strongly dependent on the probability of transmission, clearance rate and assortativeness by age than on immune response patterns and assortativeness by sexual activity. A description of sensitivity analyses [32] is provided in the Materials and Methods section, while detailed results of univariate and bivariate analyses of the relationship between parameters and log-likelihood are shown in the Supplementary Materials (Figure S2.9-S2.14 in File S7).

**Table 2:** Sensitivity analysis, by HPV type and country.

		Sensitivity Index**^*¶*^**			
		HPV16		HPV18	
Parameter		Italy	Sweden	Italy	Sweden
Probability of transmission		0.481	0.588	0.641	0.581
Rate of clearance (r(t))*	a	0.23	0.002	0.003	0.003
	b	0.07	0.068	0.07	0.07
	c	0.15	0.186	0.19	0.19
	r(t) *	0.45	0.256	0.263	0.263
Assortativeness	Age	0	0.136	0.136	0.136
	Sexual activity	0.003	0.007	0.007	0.007
Probability of developing life-long immunity after HPV infection clearance	Boys	0.032	0.007	0.007	0.007
	Girls	0.033	0.005	0.007	0.007
Adjusted R-squared		0.21	0.16	0.16	0.16

Relative importance (sensitivity index) of input parameters of the transmission model.

^*^where *r*_*(t)*_= a*EXP(-b*t)***^c^*** and *t* is time elapsed since infection.

^***¶***^The sensitivity index of each input parameter corresponds to the proportion of the total variance attributable to each parameter in multivariable quadratic regression model, where each set of input parameters of the transmission model was treated as a vector of independent variables and the log-likelihood, measuring the fit between model’s estimate and observed data, acted as dependent variable. The sensitivity index of each parameter takes values between 0 and 1. The higher is the sensitivity index the more influent is the parameter on the fit between transmission model estimates and observed data.

Abbreviation: HPV = human papillomavirus

### Projection of HPV16 prevalence among women negative three years before

Figure 4 shows a) the age-specific HPV16 prevalence at enrolment observed in the NTCC study, b) the prevalence observed three years later in the same study in a sample of women negative at baseline and c) the model-based projection of the same curve. As expected, the latter age-specific prevalence of HPV16 infection was lower than the baseline prevalence. For almost all age groups the projected prevalence fell within the 95% intervals of the observed HPV16 prevalence observed three years later among women negative at baseline. For younger age groups (i.e. 25-34) our projection tended to slightly underestimate the observed prevalence while for women older than 40 our projection slightly overestimated the observed prevalence. The observed curve shows a dip at age 35-39 which we hypothesize is related to low-risk sexual behaviour, such as decrease in concurrent partnerships, in specific periods of life (e.g. 35-39 years of age). This dip was not present in the model projection, as our model cannot explicitly account for these aspects of sexual behaviour (see Discussion). 

## Discussion

Our estimates of rate of clearance, probability of infection transmission, and pattern of immune response following clearance of HPV16 infection were highly consistent between Sweden and Italy, despite the two populations we used to draw our estimates differed substantially in terms of sexual behaviour, age distribution and study setting (screening for cervical cancer or *Chlamydia trachomatis* infection) [15,22]. Furthermore, in the Italian population our model could accurately predict HPV16 prevalence observed three years later among women negative at enrolment in the NTCC trial. The latter observation represents, in our view, a very strong validation because it shows that estimates based on baseline data were able to accurately predict what actually happened later in the cohort.

Consistency of the estimates of biological parameters in Italy and Sweden was found also for other HPV types. In particular, the probability of infection transmission, the most important determinant of the fit between observed data and model-based estimates, was consistent for HPV31, 45, 58, and 68. For types 18 and 51, the difference of our estimates of the probability of transmission in the two countries did not exceed 10%. For all HPV types the rate of clearance was consistently found to decrease less than exponentially as a function of time elapsed since infection. However, for some HPV types (31, 33, and 58) only two of the three parameters determining the exact rates of clearance were entirely consistent.

The fit between models’ outputs and observed data was assessed using a likelihood-based method, which accounts for the absolute number of type-specific HPV infections recorded in our datasets. Therefore, it is plausible to assume that some inconsistencies (e.g., in the probability of transmission and parameters determining the rate of clearance) of relatively rare hrHPV types may be due to chance. The differences between age-specific HPV prevalence in Sweden and Italy (see Figure S2.15-S2.18 in File S8), allow us to rule out that the observed consistency between HPV-related estimates in the two countries was simply due to an identical age-specific profile of hrHPV infections. 

The findings of sensitivity analyses, i.e., the dependency of the fit of hrHPV type-specific models from the variation of the same parameters in both countries, also support the validity of our models. Sensitivity analyses for HPV16 and 18 showed the strong influence of transmission probability and, to a lesser extent, of clearance rates and assortativeness by sexual activity. HrHPV clearance rates in the present model were allowed to remain constant or decrease as a function of the time that had elapsed since infection. 

The rates of clearance of all HPV types were found to decrease in both countries less than exponentially over time. Empirically observed clearances are limited by difficulties in determining the exact time of start and clearance of infections because their presence can only be observed at the time of a test. However, they also report a similar pattern [33–35]. Furthermore, we found that HPV16 and 18 types tended to persist longer than other hrHPV types, such as HPV68, 33, or 35. Although some published models allow the rates of clearance of HPV infection to vary by age [13,21] or by presence of cervical lesions [16,20], to the best our knowledge, no previous models explicitly accounted for the variation of clearance rates overtime elapsed since infection. Failing to incorporate such a time dimension in clearance rates can lead to underestimate the spontaneous HPV clearance of vaccine-targeted HPV types and, hence, overestimate the impact of vaccination programmes.

Transmission probabilities estimated for the majority of hrHPV types were compatible although those obtained from Sweden were slightly higher and more precise than those obtained from Italy. Transmission probabilities in our study are also consistent with the highest estimates obtained from other models [15,21]. Two modelling studies reported the probability of infection separately by hrHPV type [15,22]. Most of the reported values fall within our estimated IQRs, with the exception of HPV35. Estimates of the probability of HPV transmission based on empirical data have been seldom reported. Based on the pattern of concordant and discordant HPV infections within recently formed couples, Burchell et al. [36] estimated a 42% (95% CI: 36% - 47%) overall per-partnership probability of HPV (any type) transmission increasing to 68% among couples sexually active for 5–6 months, which is consistent with our estimates. Based on intensive follow-up (median interval between visits of 5.5 months), the cumulative probability of transmission over a 6-month period was estimated to be remarkably lower: 20% (95% CI: 16%–24%) [37]. However, infections that cleared during follow-up intervals did not contribute to the transmission process for the entire period (clearance of infection by index partner). In addition, plausibly some infections were transmitted and cleared during intervals, therefore not observed (clearance of infection by non-index partner). Both events would result in an underestimate of transmission probability. 

Our present findings support the hypothesis that infection clearance is followed by partial protection against re-infection with the same hrHPV type. This is however type-dependent: some 70% to 80% of HPV16 infections in both sexes clear without developing protective immunity while for most other hrHPV infections, the corresponding percentage is around 50%. Findings from women enrolled in vaccination trials also favour a partial immune protection pattern [38–40]. Vaccination was shown to reduce HPV16 or 18 re-infections among women HPV-seropositive but DNA-negative for the same type [39]. Reduced risk of re-infection was also observed in the control arms among HPV16 or 18 seropositive, DNA-negative compared to sero- and DNA-negative women [38,40]. An underestimation of protective immunity after clearance can lead to a substantial overestimation of the ultimate efficacy of vaccination programmes. 

Strengths and limitations of our present model should be born in mind. By focusing solely on the natural history of hrHPV infections we avoided the uncertainties related to the natural history of precancerous cervical lesions and cancers and could use a relatively simple model. Access to two very large datasets of prevalent HPV infections allowed us robust testing of the consistency of HPV-related estimates in two substantially different populations. The extent, to which our findings from high-income populations may also apply to middle-/low-income countries, notably those where there is no screening activity or HIV infection is frequent, i**s** unclear.

The true probabilities of infection transmission and clearance rates may differ by sex, but sufficient data from male populations are not available. We constrained the natural history of infection to be the same among men and women as in several previous studies [13,15,16,20,22,24–26,41,42]. Similarly, we did not account for the loss of natural induced immunity (SIRS model) [15,22] and the immune response following repeated infections with the same type [20]. Lack of information prevented us from accounting for a possible loss of immune response. Several potentially influential aspects of sexual behaviour, such as sexual networks, duration and overlapping of sexual partnerships, and the contribution of men-who-have-sex-with-men to the circulation of HPV infection in the general population were not explicitly accounted for in our model. However, the simplified way we used to represent sexual activity in our model has been extensively adopted in previous studies of sexually transmitted infections [43,44]. 

Neither Italian nor Swedish survey on sexual behaviour collected data from women younger than 18 years of age. Therefore, for women aged 15-17, we assumed the same sexual activity rates than the ones observed in the nearest age group. The Swedish study population included women, who voluntary attended screening for a sexually transmittable disease, thus we assumed they were sexually active. However, since we were unsure about what fraction of women aged 15-17 were sexually active, in the Swedish general population, we re-estimated the parameter values by fitting our model-based projections to type-specific HPV prevalence among women aged 18-44 years. The resulting estimates were highly consistent with those obtained from the entire population (data not shown). 

Finally, we speculate that the dip of HPV16 prevalence at age 35-39 may be related to age-dependent variations in sexual behaviour, which cannot be captured using our model. In particular, we hypothesize that the observed dip is related to a decrease in concurrent partnerships at 35-39 years of age. 

In conclusion, we provided type-specific validated estimates of biological parameters governing hrHPV natural history, which by far cannot be easily inferred from empirical data but, among other uses, are essential to forecast the impact of HPV vaccination programmes. 

## Materials and Methods

### Ethics Statement

The NTCC trial was conducted within organized cervical cancer screening programmes in nine centres in Northern and Central Italy (Turin, Trento, Padua, Verona, Florence, Bologna, Imola, Ravenna and Viterbo). All participants provided written informed consent. The study was approved by the ethics committee of the coordinating centre in Turin (Commissione sperimentazioni cliniche della Regione Piemonte - Comitato etico di riferimento regionale, art. 7, DM 18 marzo 1998) and by local ethics committees of each participating centre. 

For Sweden, the study population was from the Skåne region in Southern Sweden. All samples were anonymized. The ethical review board in Lund, Sweden, decided that informed consent was not required.

For the present modeling study, age-specific prevalences from Sweden and Italy were estimated using anonymized and aggregated data. 

### Observed Data

Data from Italy were obtained from NTCC trial [27]. A total of 47,369 women were randomly assigned to the HPV testing arm between March 2002 and December 2004. After exclusion of 469 women who did not have any valid HPV test, 46,900 women were available. A large sample of 7619 women who were HPV-negative at baseline was re-tested for HPV at the subsequent screening round, about three years later. Data from Sweden were obtained from the voluntary *Chlamydia trachomatis* screening [28]. A total of 33,137 samples were collected between March and November 2008. We included only women aged 15-44 years for whom cervico-vaginal cell samples were available and excluded urine-only samples. A total of 20,883 samples could be included. Since samples were anonymized it is not known which samples are repeated tests on the same woman. However, the number of women was estimated to be 78% of the number of samples [28,30].

### Model

In transmission models, HPV infection dynamics is determined by underlying sexual contact patterns and by the assumptions made about the natural history of HPV infection, including the relevant stages, the parameters that regulate the transition between them and their values [11]. Despite a common methodological background, the models of hrHPV (mostly, types 16 and 18) proposed in the scientific literature differ considerably between each other in terms of assumptions about hrHPV/cervical cancer natural history and of parameterization and validation methods (see Table S3.1 in File S9) [11–16,20–22,24,41,42]. .

The current biological uncertainties about immune response patterns following hrHPV infections clearance are reflected in published models [12–15,19–21,41,42]. Clearance of infections was alternatively assumed to be followed by a) the development of a natural protective immune response, i.e. SIR model [12,14]; b) lack of natural protective immune response, i.e. SIS model [13,41]; c) partial protection against re-infection with the same hrHPV type, which can be modelled assuming either various combinations of SIS and SIR models (SIS/SIR) [19–21,42] or loss of acquired immunity, i.e. SIRS model [15,22]. Assumptions about clearance patterns may substantially affect the estimates of HPV infection transmission probability and of vaccination impact [19]. All other things being equal, the beneficial impact of vaccination will be larger the more the protection from vaccination exceeds that from natural immunity [11]. Both Italian and Swedish populations were virtually unvaccinated at the time of sampling, thus we did not consider a separate compartment for vaccinated women.

Our study was conceived as two independent sets of simulations, blind to each other outputs, and based on the same transmission model and methodology. The model’s outputs were independently fitted to the hrHPV type- and age- specific prevalence (5-year age-groups, between 25 and 59 years of age for Italy and between 15 and 44 for Sweden) [22] observed in Italy and Sweden. For Italy only the prevalence at recruitment was used.

The age-structured dynamic model of the transmission of individual-HPV-type-infections used in the present study (see File S1) is an evolution of an earlier version [19,24]. Briefly, the following modifications have been introduced: a) the model only considers infection transmission dynamics and does not deal with the progression from infection to pre-cancerous lesions and cancer; and b) we allowed for different immune response after infection clearance, i.e. SIS, SIR or SIS/SIR model (SIRS model was not contemplated). Cervical intraepithelial neoplasia (CIN) lesions are a result of persistent infection but could also represent a cause of persistence. We did not explicitly model CIN dynamics, but this is implicitly accounted for in our estimates of the rates of infection clearance, which decreases with increasing persistence. Screening could have led to an over-estimate of “natural” infection clearance by removing CIN. However, as CIN lesions represent a very small fraction of HPV infections, bias should be very small. For example the detection rate of CIN2+ in Italy (3.2 per 1000 screened women [45]) was about one order of magnitude lower than HPV infection prevalence. 

Our models use partial integro-differential equations solved with respect to calendar time, age, and time elapsed since HPV infection (infection duration). Rates of clearance of hrHPV infections were allowed to change according to the time elapsed since infection [33–35]. Equation 1 describes the variation of hrHPV clearance rates (r) as a function of time elapsed since infection (*t*)

(Eq. 1) $$r_{\left(t\right)}=a\cdot exp^{{\left(-b\cdot t\right)}^{c}}$$

where *a* is the rate of clearance at the acquisition of infection, *b* is the decrease of the clearance rate *a* overtime *t*, and *c* is a time effect modifier with respect to exponential decrease. For example, if *c* = 0 then *r* is constant over time; if *c* = 1 then *r* is decreasing exponentially over time, if *c*> 1 then *r* is decreasing more than exponentially, whereas c < 1 then *r* is decreasing less than exponentially over time. The full mathematical description of the model is reported in the Supporting Information section. Given the clear evidence that infections with different carcinogenic HPV types behave differently but independently from each other [46–48], 13 individual hrHPV types have been modelled separately, assuming no interaction between types.

### Sexual behaviour

For both countries, information on sexual behaviour was collected from nation-wide population-based surveys [29,30] and applied to study populations. We represented the network of sexual partnerships by making some simplifications about contact patterns within the two populations [31,49]: a) all sexual contacts were heterosexual; b) concurrent sexual partnerships are not explicitly accounted for; and c) the annual rate of acquisition of new sexual partners only varied by age-group (5-year age-groups ranging between 14 and 75 years) and CSA (two and three for Sweden and Italy, respectively). Sexual assortativeness by age and CSA were estimated by fitting the model’s outputs to the observed data. For comparability, we used the same sexual activity parameter values reported in previous publications [22,24]. Death rates were obtained from the respective National Statistical Institutes and the age distribution of the two populations was assumed to remain constant over time (Tables S1.1 & S1.2 in File S2). 

### Model fitting and validation

To fit our model estimates to country-specific observed data we adapted the method proposed by Van de Velde et al [21]. Briefly, 100,000 sets of parameter values were generated by independently sampling, for each parameter, a uniform distribution within a pre-specified range of values, using a Latin hypercube algorithm [32]. The ranges of values explored for each parameter are reported in Tables S2.1, S2.2 & S2.3 in File S3. Each set of values was used to generate a model-based age-specific curve of prevalence for each hrHPV type. Finally, each model’s output was compared to the above-mentioned observed age-specific prevalence of each hrHPV, by calculating binomial log-likelihood. To account for differences in sexual behaviour between the two populations we fitted to observed data from Italy and Sweden the outputs of the model obtained for a) all CSAs combined and b) the highest CSA, respectively. 

We selected the 100 model-generated curves that fitted best the observed data (Figure S2.2-S2.4 in File S5, S2.5-S2.8 in File S6) and among them we computed, for each parameter, the median and IQR values as estimates of the most credible parameter values. Estimates of biological parameters were provided for each hrHPV type separately. Conversely, the same estimates of assortativeness by age and CSA were used for all hrHPV. We compared the distribution of the set of 100 best parameter-specific estimates obtained in Sweden and Italy by the Mann-Whitney test, as α–level we considered both 0.05 and 0.01. In addition, we have compared the HPV16 age-specific prevalence observed three years later among initially negative women in the Italian study with the model-based projection of the same curve. Since our model accounts for time elapsed since infection, we estimated the HPV16 age-specific prevalence as the ratio of women with an infection not older than 3 years to women susceptible to infection 3 years before. We restricted this analysis to HPV16 because the samples available for other types were insufficient.

### Sensitivity analyses

For HPV16 and 18, we assessed (across the best 10,000 fitting estimates) the sensitivity of the fit of model’s estimates to the variation of input parameters according to the method proposed by Hoare et al. [32]. Briefly, we defined a multivariable quadratic regression model where each set of input parameters of the transmission model was treated as a vector of independent variables and the log-likelihood, measuring the fit between model’s estimate and observed data, as dependent variable. To assess the relative contribution of each parameter to a good model fit we calculated a sensitivity index. Its value can range between 0 and 1 and represents the proportion of the total variance of the log-likelihood attributable to each parameter in each country. Latin hypercube sampling and sensitivity analyses were performed using the Sampling and Sensitivity Analyses Tools (SaSAT) for computational modelling [32]. 

## Supporting Information

File S1 **Model structure: figure and equations.**
(PDF)

File S2 **Tables S1.1 & S1.2.** Assumed behavioural and demographic parameters.
(PDF)

File S3 **Tables S2.1, S2.2 & S2.3.** Estimated parameters.
(PDF)

File S4 **Figure S2.1.** Estimated median cumulative probability (%) of HPV infection clearance, by country.
(PDF)

File S5 **Figures S2.2-S2.4.** Fit between prevalence curves and model outputs by country-Part A.
(PDF)

File S6 **Figures S2.5-S2.8.** Fit between prevalence curves and model outputs by country- Part B.
(PDF)

File S7 **Figures S2.9-S2.14.** Results of univariate and bivariate analyses.
(PDF)

File S8 **Figures S2.15-S2.18.** Type-specific HPV prevalence curves by country.
(PDF)

File S9 **Table S3.1.** Summary of published models.
(PDF)
