# Supplementary material for: Type-Specific Human Papillomavirus Biological Features: Validated Model-Based Estimates
Source: PLoS One. 2013 Nov 29;8(11):e81171. doi: 10.1371/journal.pone.0081171 (PMC3882251; doi:10.1371/journal.pone.0081171)
Supplement: File S1 — Model structure: figure and equations. (PDF) [file pone.0081171.s001.pdf]

Figure S1.1. Single-type HPV transmission model. The dynamics of the infection transmission accounts for calendar time, women's age, and time elapsed since infection (or infection duration). Note that the dimensions of the arrows connecting the different compartments are not proportional to the rates of transition. For the model's details, see Supplementary Materials section below.

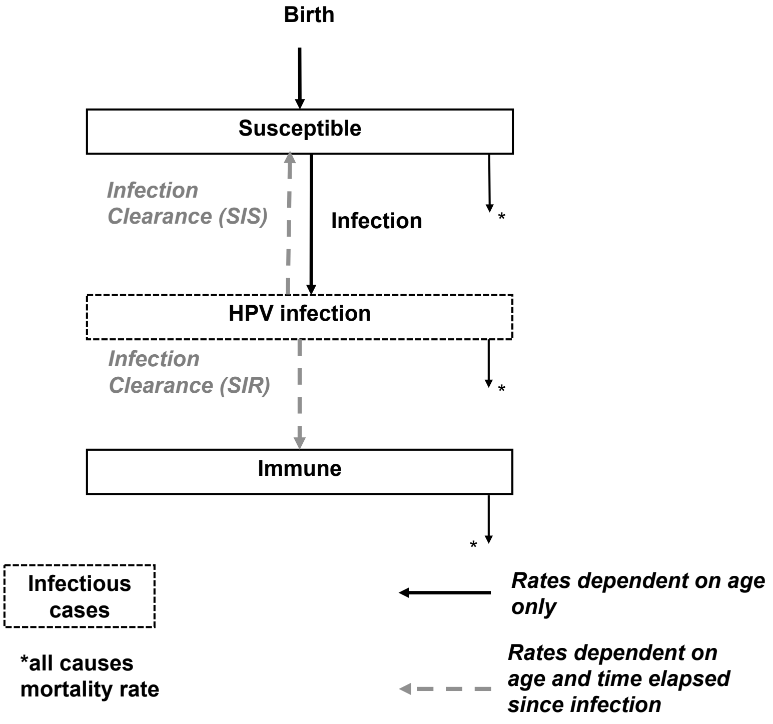

SIS: susceptible-infected-susceptible; SIR susceptible-infected-recovered/immune.

Model's equations

Legend

k : gender of interest (women and men)
l : class of sexual activity (CSA) of the gender of interest
m : CSA of the opposite gender
i : age class of the gender of interest
j : age class of the opposite gender

t: calendar time
a: age
t_I:time elapsed since infection (TSI)

S: Susceptible sexually active population (stratified by gender, age, and CSA)
I: Type specific HPV infected/infectious (stratified by gender, age, CSA, and TSI)
R: Type specific HPV immune (stratified by gender, age, and CSA)

m: Death Rate (stratified by gender and age)

pW: Proportion of Women at birth (0.5)
pM: Proportion of Men at birth (0.5)
CSAprop: Proportion in a specific Class of Sexual activity (stratified by gender and age)

l: Force of Infection or per-capita annual rate of infection (stratified by gender, age, and CSA)
c: Per-capita annual rate of clearance of infection (stratified by TSI)
a: Proportion of infections clearing by developing protective immunity (stratified by gender)

Boundary Conditions

Calendar time

Susceptible: $S_{k\_l}(a,0)$

HPV infected/infectious: $\bar{I}_{\_l}(a,0) = \int_{0}^{\infty} I_{\_k}(a,0,\tau_1)\cdot d\tau_1$

Immune: $R_{k\_l}(a,0)$

Total(Women): $N_{k\_l}(a,0) = S_{k\_l}(a,0) + \bar{I}_{\_k}(a,0) + R_{k\_l}(a,0)$

Age

$$S_{k\_l}(14^*,t) = 0.5\cdot CSAprop_{k\_l}\cdot \mu_k(a)\cdot\left[S_k\left(a,t\right) + R_k\left(a,t\right) + \int_{0}^{\infty} I_{\_k}(a,0,\tau_1)\cdot d\tau_1\right]$$

*Sexual debut assumed at age 14yrs

$$N_{tot}(t) = N_{women\_tot}(t) + N_{men\_tot}(t)$$
$$0 = I_{\_l}(14,t,\tau) = R_{w\_l}(15,t,\tau)$$

Time elapsed since infection (TSI)

$$I_{k\_l}(a,t,0) = \lambda_{k\_l}(a,t)\cdot S_{k\_l}(a,t)$$

Dynamic model system

1. $$\frac{\partial S_{k\_l}\left(a,t\right)}{\partial a} + \frac{\partial S_{k\_l}\left(a,t\right)}{\partial t} = \int_{0}^{n}\left(1-\alpha_k\right)\cdot\chi(a,\tau_1)\cdot I_{k\_l}(a,t,\tau_1)d\tau_1 - [\lambda_{k\_l}(a,t) + \mu_k(a)]\cdot S_{k\_l}(a,t)$$

2. $$\frac{\partial I_{k\_l}\left(a,t,\tau_1\right)}{\partial a} + \frac{\partial I_{k\_l}\left(a,t,\tau_1\right)}{\partial t} + \frac{\partial H_{k\_l}\left(a,t,\tau_1\right)}{\partial \tau_2} = -\left[\chi_k(a,\tau_2) + \mu_k(a)\right]\cdot I_{k\_l}\left(a,t,\tau_1\right))$$

3. $$\frac{\partial R_{k\_l}\left(a,t\right)}{\partial a} + \frac{\partial R_{k\_l}\left(a,t\right)}{\partial t} = \int_{0}^{n}\alpha_k\cdot\chi(a,\tau_1)\cdot I_{k\_l}(a,t,\tau_1)d\tau_1 - \mu_w(a)\cdot R_{w\_l}(a,t)$$

Force of infection

Indices	Definition
ε_{age}	Mixing according to age (ε_{age}=1 for random and ε_{age}=0 for assortative)
ε_{csa}	Mixing according to sexual activity (ε_{csa}=1 for random and ε_{csa}=0 for assortative)
δ_{ij}	Identity matrix for age (δ_{ij}=1 if i=j and δ_{ij} =0 if $i\neq j$)
δ_{lm}	Identity matrix for age (δ_{lm}=1 if l=m and δ_{lm} =0 if $l\neq m$)
age	Age groups (see table S2)
csa	Class of sexual activity
$c^*_{k\_lmij}(t)$	the mean rate (year^{-1}) at which someone of gender k of sexual activity class l and age i acquires a new sexual partner
$\rho K_{lmij}(t)$	the conditional probability that the sex partner of someone of gender k , sexual activity class l, and age i, is in the sexual activity class m, and age class j of the opposite gender k'.
β_k	the transmission probability (per partnership) from someone of gender k' to susceptible partner of gender k
θ	is the degree to which men and women alter their behaviour

Susceptible individuals ($S_{k'\_il}(t)$) are infected at an age (i) and sexual activity (l) specific rate per year; the force of infection ($\lambda_{k'\_il}(t)$).
The force of infection,

$$\lambda_{k'\_il}(t)=\sum_{j=1}^{n1}\sum_{m=1}^{n2}\left[c^*_{k'\_lmij}(t)\cdot\rho K'_{lmij}(t)\cdot\left(\frac{\sum_{\tau_1=1}^{n3}\beta_k(a,\tau_1)\cdot I_{k\_mj}(a,t,\tau_1)}{N_{k\_mj}(a,t)}\right)\right]$$

is a function of the likelihood per sexual partnership that HPV is transmitted from an infected to a susceptible partner (β_k), the rate of sexual partner acquisition ($c_{k\_il}(t)$, the fraction of these partners infected and the pattern of mixing between sex partners according to sexual activity ($\rho K_{lmij}(t)$)

$$\rho K_{lmij}(t)=\left[\varepsilon_{age}\frac{\sum_{csa=1}^{a}(N_{k'\_csa\_j}(t)\cdot c_{k'\_csa-j}(t))}{\sum_{csa=1}^{a}\sum_{age=1}^{b}N_{k'\_csa\_age}(t)\cdot c_{k'\_csa\_age}(t)}+(1-\varepsilon_{age})\delta_{ij}\right]\cdot\left[\varepsilon_{csa}\frac{N_{k'\_csa\_age}(t)\cdot c_{k'\_csa\_age}(t)}{\sum_{csa=1}^{a}(N_{k'\_csa\_j}(t)c_{k'\_csa\_j}(t))}+(1-\varepsilon_{csa})\delta_{lm}\right]$$

There can be discrepancies in the number of new partners reported for men and women, thought to be due in part to underreporting by women. If there is a discrepancy between the rate of sexual partner formation of one sex, age and activity group and the reciprocal group of the other sex, age and activity group, then the rate of sexual partner change of both sexes is adjusted so that the rates match. The discrepancy in rate of sexual partner formation (Balance_{lmij}(t)) is calculated by the equation:

$$Balance_{lmij}(t) = \frac{N_{k\_il}(t) \cdot c_{k\_il}(t) \cdot \rho K_{lmij}(t)}{N_{k'\_il}(t) \cdot c_{k'\_il}(t) \cdot \rho K'_{lmij}(t)}$$

The discrepancy adjusted contact rate for women in each age (i) and sexual activity (l) class with men in each age (j) and sexual activity (m) class is:

$$c^{*}_{k'\_lmij}(t) = c_{k'\_il} \cdot Balance_{lmij}(t)^{theta}$$

The partner change rate for men adjusted in the reverse direction becomes:

$$c^{*}_{k\_lmij}(t) = c_{k\_il} \cdot Balance_{lmijl}(t)^{-(1-theta)}$$

For this model it was assumed that men and women alter their behaviour equally (i.e. θ_1=0.5).

The methods used are similar to those previously used in modelling HIV and HPV transmission dynamics (*16, 31, 49*). Using patterns of sexual mixing as the basis for disease transmission allows the influence of changes in behaviour on disease dynamics to be explored.
