# Supplementary material for: Type-Specific Human Papillomavirus Biological Features: Validated Model-Based Estimates
Source: PLoS One. 2013 Nov 29;8(11):e81171. doi: 10.1371/journal.pone.0081171 (PMC3882251; doi:10.1371/journal.pone.0081171)
Supplement: File S2 — Tables S1.1 & S1.2. Assumed behavioural and demographic parameters. (PDF) [file pone.0081171.s002.pdf]

Table S1.1. Sexually activity parameter values imposed to the modelled population, by country

Class of sexual activity (% of population)	Italy			Sweden			
	Both Genders			Men		Women	
	Low 77%	Medium 18%	High 5%	Low 85%	High 15%	Low 85%	High 15%
Age							
14–19	0.34	2.34	5.67	0.54	4.11	0.25	2.89
20–24	0.33	2.32	13.46	0.54	4.11	0.25	2.89
25–29	0.44	2.66	9.22	0.54	4.11	0.25	2.89
30–34	0.44	2.60	7.23	0.05	0.64	0.07	1.47
35–39	0.47	2.54	5.87	0.05	0.64	0.07	1.47
40–44	0.47	2.37	4.97	0.05	0.64	0.07	1.47
45–59	0.46	2.39	6.60	0.05	0.64	0.07	1.47
60-75	0.00	0.00	0.00	0.01	0.21	0.02	0.11

Table S1.2. Mortality rates (per 1,000) imposed to the modelled population, by country

Age	Italy		Sweden	
	Men	Women	Men	Women
<20	0.9	0.32	0.267	0.166
20–24	1.16	0.36	0.727	0.258
25–29	1.15	0.46	0.707	0.264
30–34	1.27	0.58	0.712	0.357
35–39	1.65	0.8	0.842	0.458
40–44	2.35	1.26	1.483	0.909
45–49	3.97	2.14	1.919	1.229
50–54	6.91	3.19	3.921	2.561
55–59	12.03	5.15	5.069	3.31
60–64	19.45	8.06	10.254	6.331
65–69	28.93	13.26	13.814	8.238
70–74	46.33	23.54	29.337	16.98
75–79	74.2	42.43	39.928	23.658
