# Supplementary material for: Type-Specific Human Papillomavirus Biological Features: Validated Model-Based Estimates
Source: PLoS One. 2013 Nov 29;8(11):e81171. doi: 10.1371/journal.pone.0081171 (PMC3882251; doi:10.1371/journal.pone.0081171)
Supplement: File S3 — Tables S2.1, S2.2 & S2.3. Estimated parameters. (PDF) [file pone.0081171.s003.pdf]

Table S2.1. Explored ranges and estimated median (and inter-quartile range) of a) probability of infection transmission per sexual partnership; b) determinants of per-capita annual rate of clearance of infection; c) probability of developing life-long protective immunity after HPV clearance, by hrHPV types (Sweden).

HPV type	Probability of infection transmission per sexual partnership (beta)	Per-capita annual rate of clearance of infection (*r*)[§]			Probability of developing life-long immunity after HPV infection clearance	
		Rate of clearance at time *t0* (*a*)	Rate of decrease of clearance rate *a* (*b*)	Modifier with respect to exponential decrease (*c*)[*]	Boys	Girls
HPV 16	0.79 (0.69 - 0.86)	1.8 (1.66 - 2.03)	0.87 (0.65 - 1.13)	0.52 (0.41 - 0.65)	0.32 (0.16 - 0.54)	0.2 (0.09 - 0.32)
HPV 18	0.82 (0.73 - 0.9)	1.99 (1.75 - 2.24)	0.96 (0.64 - 1.2)	0.47 (0.36 - 0.67)	0.49 (0.23 - 0.73)	0.43 (0.23 - 0.63)
HPV 31	0.75 (0.64 - 0.84)	1.96 (1.76 - 2.23)	0.84 (0.65 - 1.13)	0.5 (0.37 - 0.65)	0.47 (0.23 - 0.68)	0.26 (0.14 - 0.45)
HPV 33	0.84 (0.77 - 0.93)	2.52 (2.19 - 2.79)	0.82 (0.63 - 1.13)	0.58 (0.44 - 0.78)	0.6 (0.34 - 0.81)	0.78 (0.57 - 0.87)
HPV 35	0.56 (0.46 - 0.67)	2.87 (2.27 - 3.25)	0.95 (0.68 - 1.18)	0.72 (0.57 - 0.99)	0.59 (0.28 - 0.8)	0.63 (0.31 - 0.84)
HPV 39	0.74 (0.64 - 0.84)	2.31 (2.04 - 2.75)	0.82 (0.63 - 1.16)	0.62 (0.5 - 0.8)	0.48 (0.22 - 0.68)	0.51 (0.35 - 0.76)
HPV 45	0.64 (0.54 - 0.75)	2.4 (1.95 - 2.88)	0.86 (0.71 - 1.08)	0.69 (0.49 - 0.87)	0.6 (0.29 - 0.8)	0.48 (0.25 - 0.73)
HPV 51	0.8 (0.72 - 0.92)	1.82 (1.63 - 2.13)	0.81 (0.64 - 1.18)	0.48 (0.34 - 0.61)	0.4 (0.19 - 0.59)	0.43 (0.27 - 0.6)
HPV 52	0.72 (0.62 - 0.83)	2.06 (1.77 - 2.41)	0.8 (0.65 - 1.04)	0.61 (0.45 - 0.78)	0.54 (0.28 - 0.72)	0.4 (0.21 - 0.66)

HPV 56	0.78 (0.72 - 0.87)	2.46 (2.04 - 2.77)	0.89 (0.64 - 1.2)	0.63 (0.46 - 0.82)	0.56 (0.25 - 0.72)	0.64 (0.39 - 0.81)
HPV 58	0.76 (0.67 - 0.87)	2.48 (2.13 - 2.75)	0.86 (0.6 - 1.17)	0.6 (0.45 - 0.81)	0.56 (0.38 - 0.8)	0.66 (0.42 - 0.85)
HPV 59	0.85 (0.75 - 0.92)	2.31 (1.88 - 2.61)	0.77 (0.61 - 1.09)	0.48 (0.28 - 0.73)	0.54 (0.33 - 0.78)	0.65 (0.43 - 0.84)
HPV 68	0.48 (0.4 - 0.59)	2.85 (2.38 - 3.18)	0.91 (0.68 - 1.2)	0.78 (0.57 - 1.06)	0.61 (0.28 - 0.78)	0.52 (0.28 - 0.82)
Explored Ranges[¶]	0.0 – 1.0	1.5 – 3.5	0.5 -1.5	0.0 – 2.0	0.0 – 1.0	0.0 – 1.0

[§] $r_{(t)}= a$*EXP(-b*t)^c. [*]$r_{(t)}$: constant if c = 0; decreasing exponentially if c = 1; decreasing more than exponentially if c > 1; decreasing less than exponentially if c < 1. [¶]100,000 different combinations of parameter values were drawn from the prior uniform distributions, using the Latin Hypercube sampling method within the explored range. The explored ranges of *a*, *b*, and *c* were based on previous work (*24*).

Table S2.2. Explored ranges and estimated median (and inter-quartile range) of a) probability of infection transmission per sexual partnership; b) determinants of per-capita annual rate of clearance of infection; c) probability of developing life-long protective immunity after HPV clearance, by hrHPV types (Italy).

HPV type	Probability of infection transmission per sexual partnership (beta)	Per-capita annual rate of clearance of infection (*r*)[§]			Probability of developing life-long immunity after HPV infection clearance	
		Rate of clearance at time *t0* (*a*)	Rate of decrease of clearance rate *a* (*b*)	Modifier with respect to exponential decrease (*c*)[*]	Boys	Girls
HPV 16	0.8 (0.67 - 0.9)	1.79 (1.63 - 2.04)	0.83 (0.66 - 1.12)	0.54 (0.42 - 0.67)	0.28 (0.14 - 0.49)	0.25 (0.14 - 0.39)
HPV 18	0.73 (0.55 - 0.85)	2.01 (1.74 - 2.39)	0.93 (0.7 - 1.16)	0.46 (0.39 - 0.62)	0.5 (0.29 - 0.72)	0.53 (0.32 - 0.74)
HPV 31	0.75 (0.57 - 0.88)	1.75 (1.63 - 1.98)	0.88 (0.67 - 1.14)	0.47 (0.36 - 0.65)	0.41 (0.18 - 0.61)	0.4 (0.27 - 0.62)
HPV 33	0.49 (0.33 - 0.74)	2.27 (1.98 - 2.6)	0.8 (0.65 - 1.09)	0.58 (0.42 - 0.74)	0.54 (0.31 - 0.8)	0.68 (0.45 - 0.83)
HPV 35	0.29 (0.21 - 0.4)	2.59 (2.14 - 3.08)	0.81 (0.59 - 1.12)	0.67 (0.5 - 0.93)	0.53 (0.3 - 0.75)	0.66 (0.34 - 0.82)
HPV 39	0.57 (0.34 - 0.75)	2.42 (2.05 - 2.68)	0.9 (0.66 - 1.12)	0.56 (0.46 - 0.74)	0.55 (0.27 - 0.75)	0.66 (0.44 - 0.87)
HPV 45	0.6 (0.43 - 0.84)	2.45 (2.09 - 2.84)	0.9 (0.74 - 1.15)	0.56 (0.47 - 0.72)	0.52 (0.3 - 0.81)	0.58 (0.35 - 0.77)
HPV 51	0.72 (0.52 - 0.89)	1.86 (1.64 - 2.2)	0.83 (0.66 - 1.11)	0.42 (0.34 - 0.54)	0.45 (0.23 - 0.68)	0.44 (0.21 - 0.67)
HPV 52	0.55 (0.29 - 0.78)	2.34 (1.99 - 2.65)	0.86 (0.68 - 1.13)	0.59 (0.46 - 0.76)	0.59 (0.34 - 0.77)	0.75 (0.5 - 0.87)
HPV 56	0.49	2.64	0.95	0.66	0.51	0.73

	(0.26 - 0.79)	(2.16 - 3.02)	(0.75 - 1.19)	(0.52 - 0.82)	(0.31 - 0.74)	(0.57 - 0.89)
HPV 58	0.74<br>(0.53 - 0.88)	2.02<br>(1.79 - 2.29)	0.93<br>(0.73 - 1.18)	0.5<br>(0.39 - 0.65)	0.63<br>(0.33 - 0.84)	0.69<br>(0.52 - 0.85)
HPV 59	0.41<br>(0.28 - 0.64)	2.48<br>(2.05 - 2.89)	0.81<br>(0.63 - 1.07)	0.63<br>(0.47 - 0.78)	0.54<br>(0.25 - 0.76)	0.45<br>(0.25 - 0.73)
HPV 68	0.42<br>(0.29 - 0.61)	2.63<br>(2.19 - 3.08)	0.85<br>(0.67 - 1.15)	0.65<br>(0.48 - 0.79)	0.62<br>(0.34 - 0.86)	0.69<br>(0.52 - 0.85)
Explored Ranges[¶]	0.0 – 1.0	1.5 – 3.5	0.5 -1.5	0.0 – 2.0	0.0 – 1.0	0.0 – 1.0

[§] $r_{(t)}$= *a**EXP(-*b***t*)^c. [*]$r_{(t)}$: constant if *c* = 0; decreasing exponentially if *c* = 1; decreasing more than exponentially if *c* > 1; decreasing less than exponentially if *c* < 1. [¶]100,000 different combinations of parameter values were drawn from the prior uniform distributions, using the Latin Hypercube sampling method within the explored range. The explored ranges of *a*, *b*, and *c* were based on previous work (*24*).

Table S2.3. Estimated median (and inter-quartile range) of sexual assortativeness by age, sexual activity, and country.

hrHPV types	Italy		Sweden	
	Age*	Sexual Activity¶	Age	Sexual Activity
All types	0.2 (0.1 – 0.4)	0.3 (0.1 – 0.7)	0.4 (0.3 - 0.6)	0.4 (0.2 - 0.7)
Explored Ranges	0.0 – 1.0	0.0 – 1.0	0.0 – 1.0	0.0 – 1.0

¥Fully and randomly assortative behaviour corresponds to value 0 and 1, respectively.

*,¶ Estimates not consistent across countries, as assessed through Mann-Whitney test (alpha-level=0.05).
