# Supplementary material for: Type-Specific Human Papillomavirus Biological Features: Validated Model-Based Estimates
Source: PLoS One. 2013 Nov 29;8(11):e81171. doi: 10.1371/journal.pone.0081171 (PMC3882251; doi:10.1371/journal.pone.0081171)

Figure S2.1. Estimated median cumulative probability (%) of HPV infection clearance, by HPV type and country.

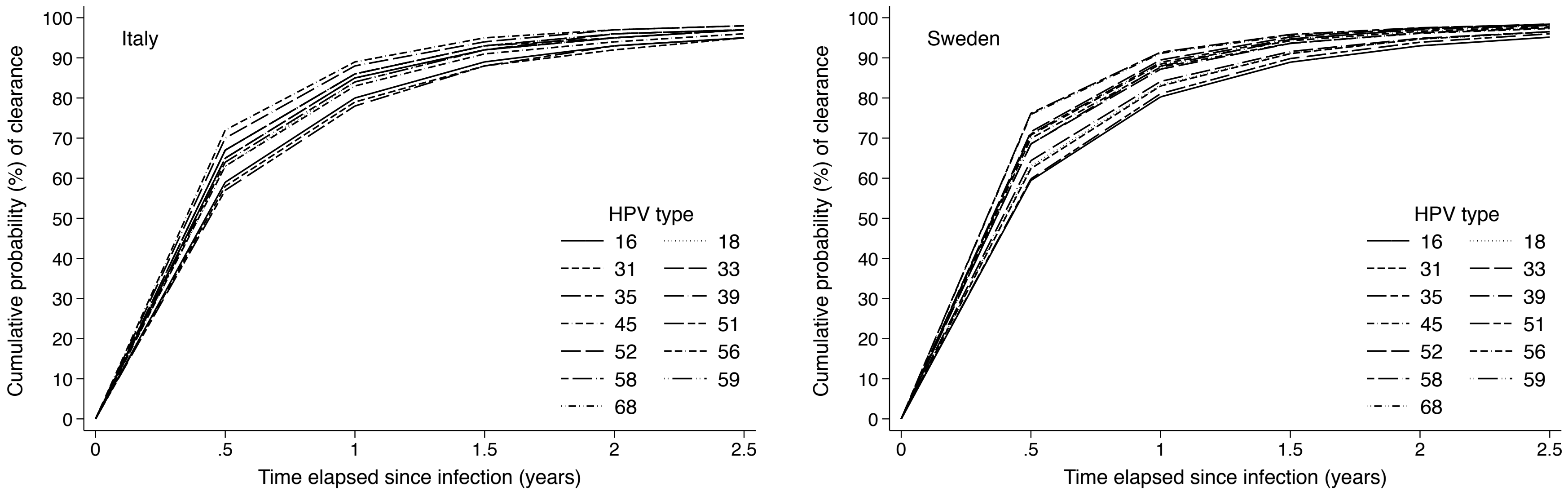

Supplement: File S4 — Figure S2.1. Estimated median cumulative probability (%) of HPV infection clearance, by country. (PDF) [file pone.0081171.s004.pdf]
