# Supplementary material for: Type-Specific Human Papillomavirus Biological Features: Validated Model-Based Estimates
Source: PLoS One. 2013 Nov 29;8(11):e81171. doi: 10.1371/journal.pone.0081171 (PMC3882251; doi:10.1371/journal.pone.0081171)

Figure S2.2. Fit between observed age-specific HPV16 and 18 prevalence and the 100 best fitting estimated curves, by country.

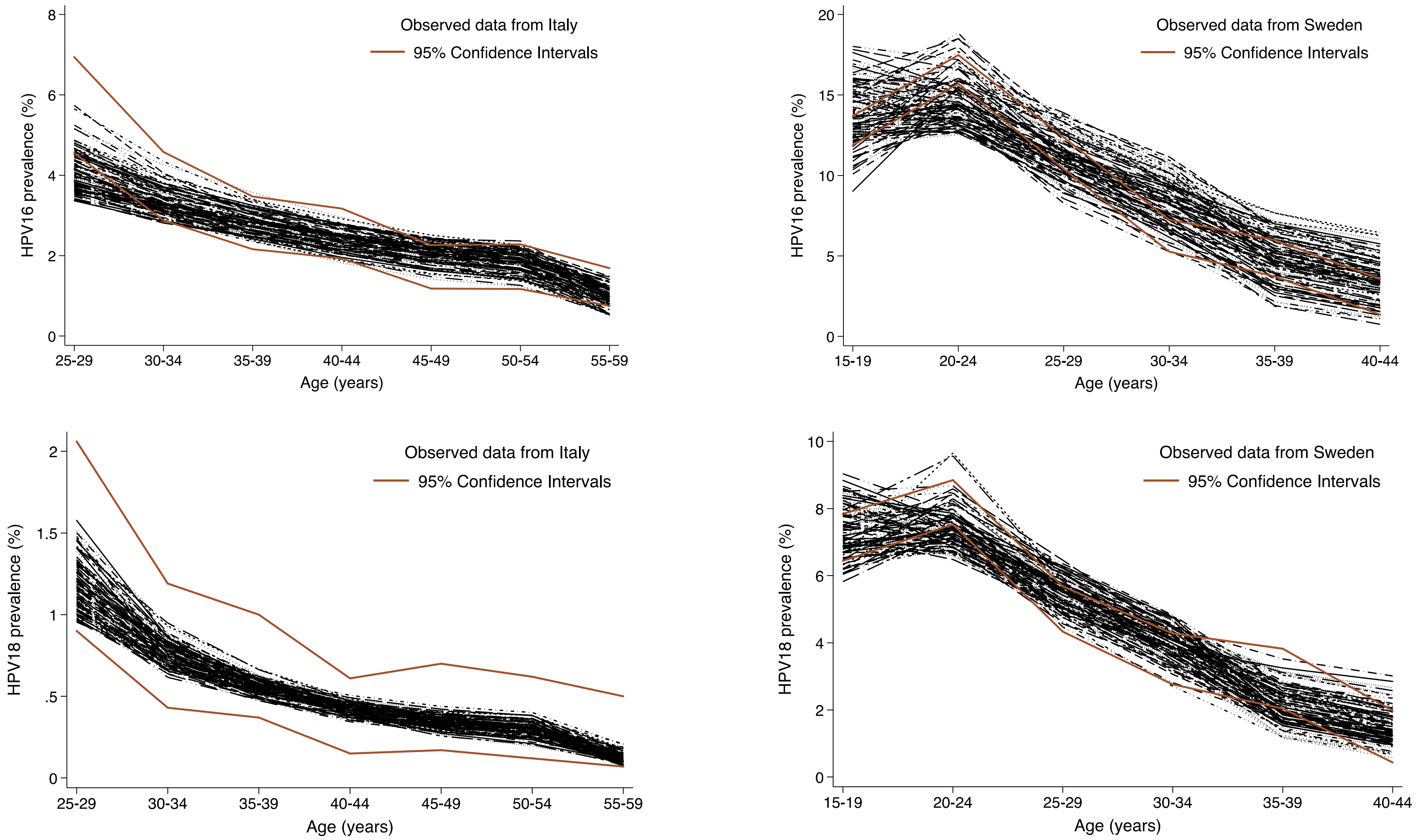

Figure S2.3. Fit between observed age-specific HPV31 and 33 prevalence and the 100 best fitting estimated curves, by country.

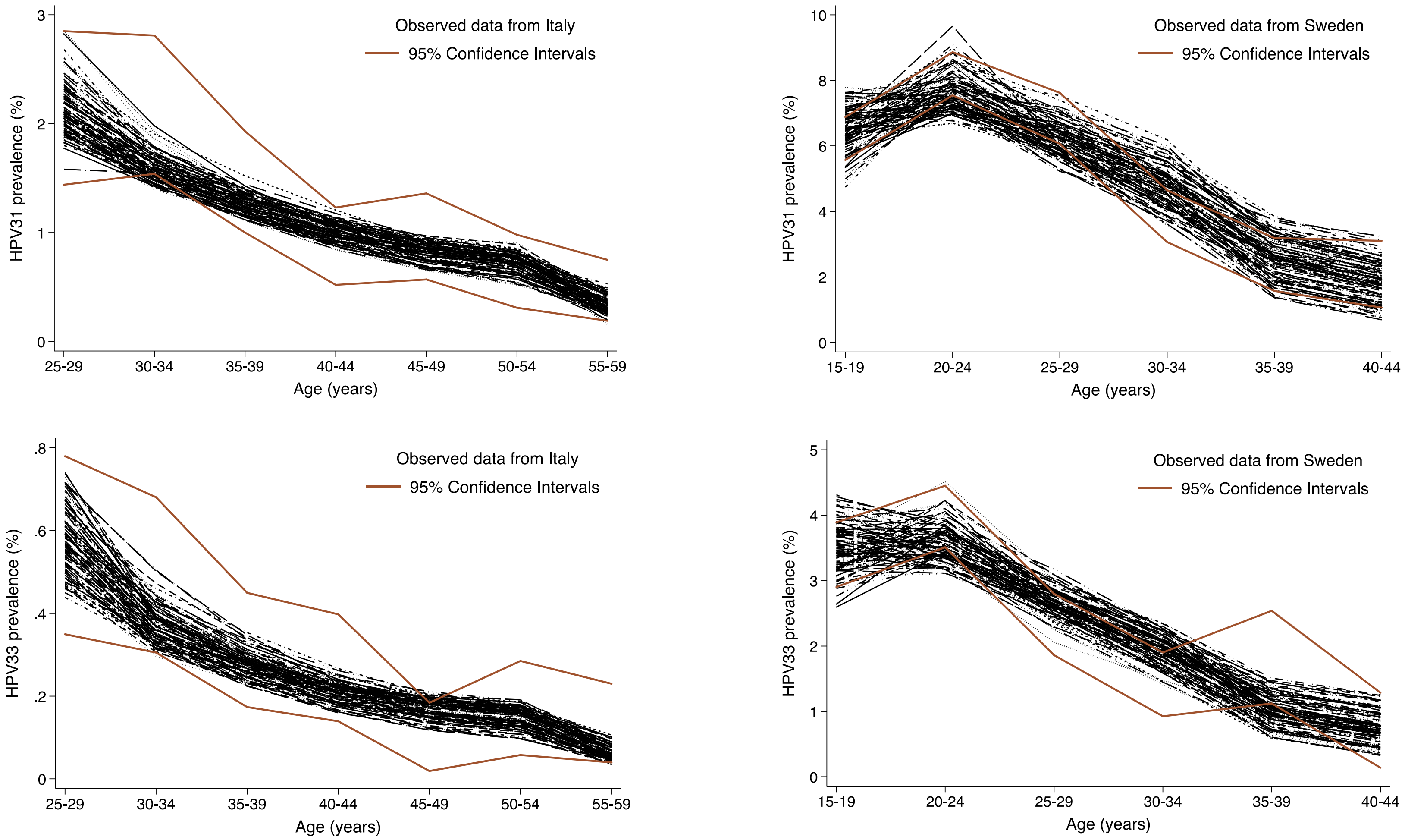

Figure S2.4. Fit between observed age-specific HPV35 and 39 prevalence and the 100 best fitting estimated curves, by country.

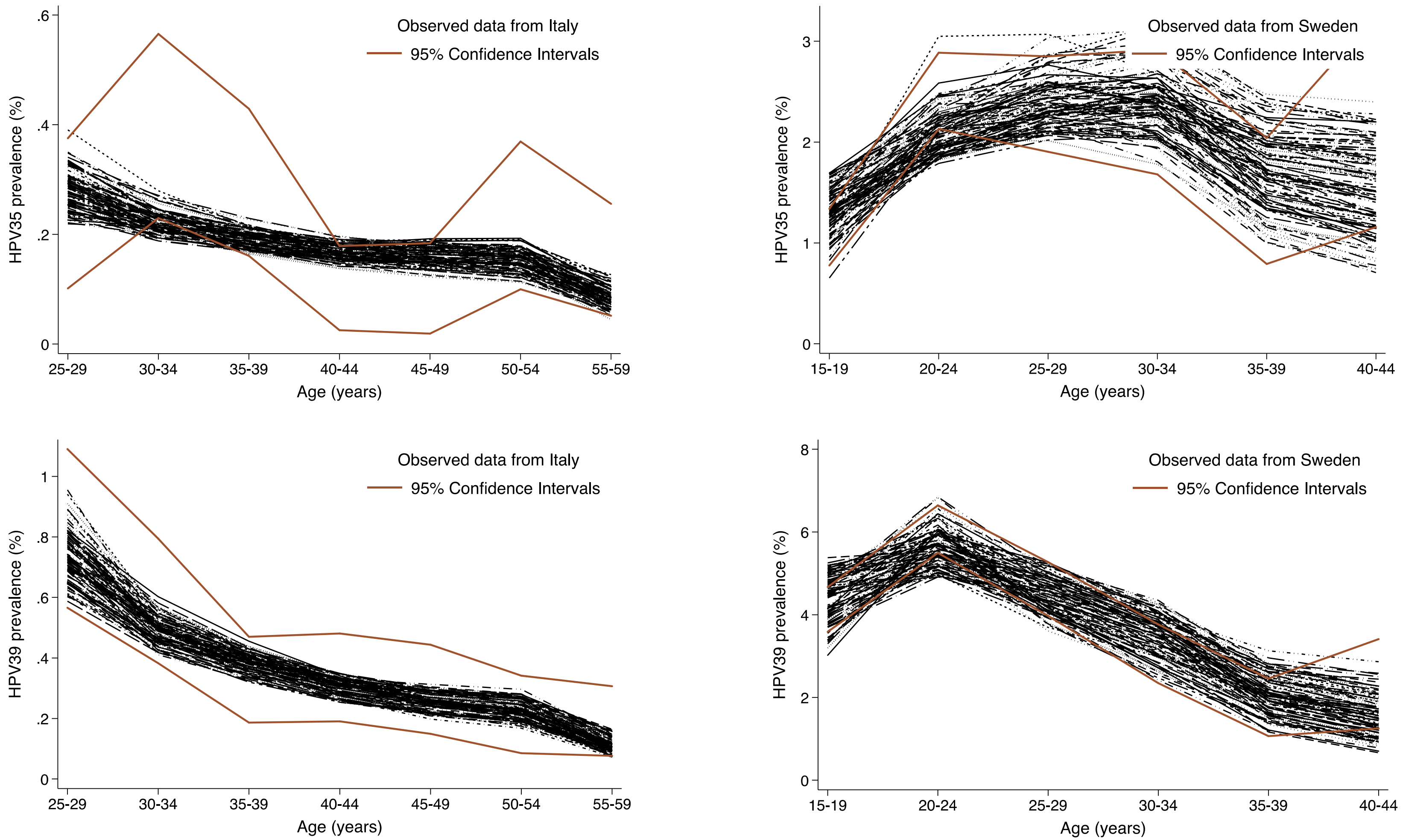

Supplement: File S5 — Figures S2.2-S2.4. Fit between prevalence curves and model outputs by country-Part A. (PDF) [file pone.0081171.s005.pdf]
