# Supplementary material for: Type-Specific Human Papillomavirus Biological Features: Validated Model-Based Estimates
Source: PLoS One. 2013 Nov 29;8(11):e81171. doi: 10.1371/journal.pone.0081171 (PMC3882251; doi:10.1371/journal.pone.0081171)

Figure S2.5. Fit between observed age-specific HPV45 and 51 prevalence and the 100 best fitting estimated curves, by country.

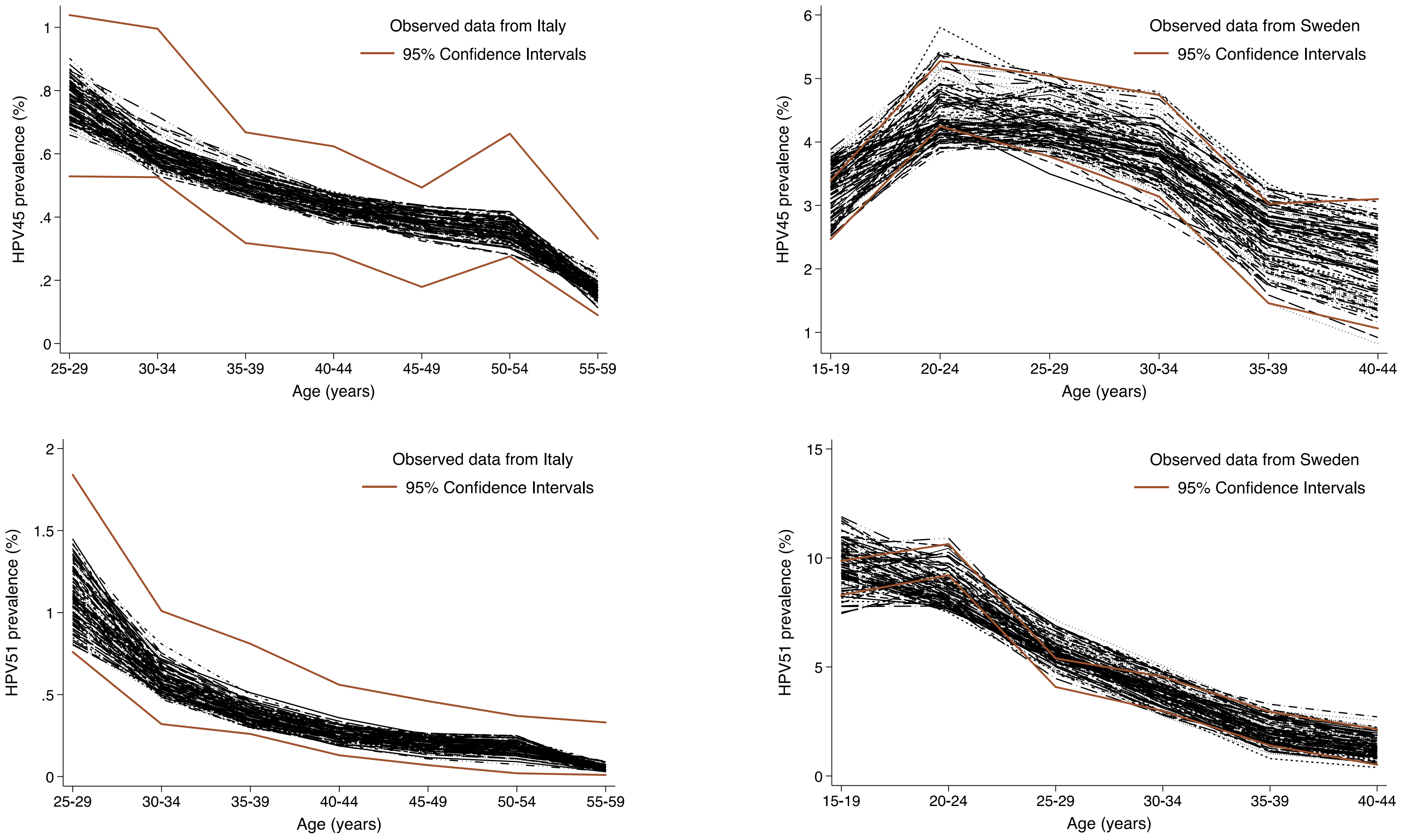

Figure S2.6. Fit between observed age-specific HPV52 and 56 prevalence and the 100 best fitting estimated curves, by country.

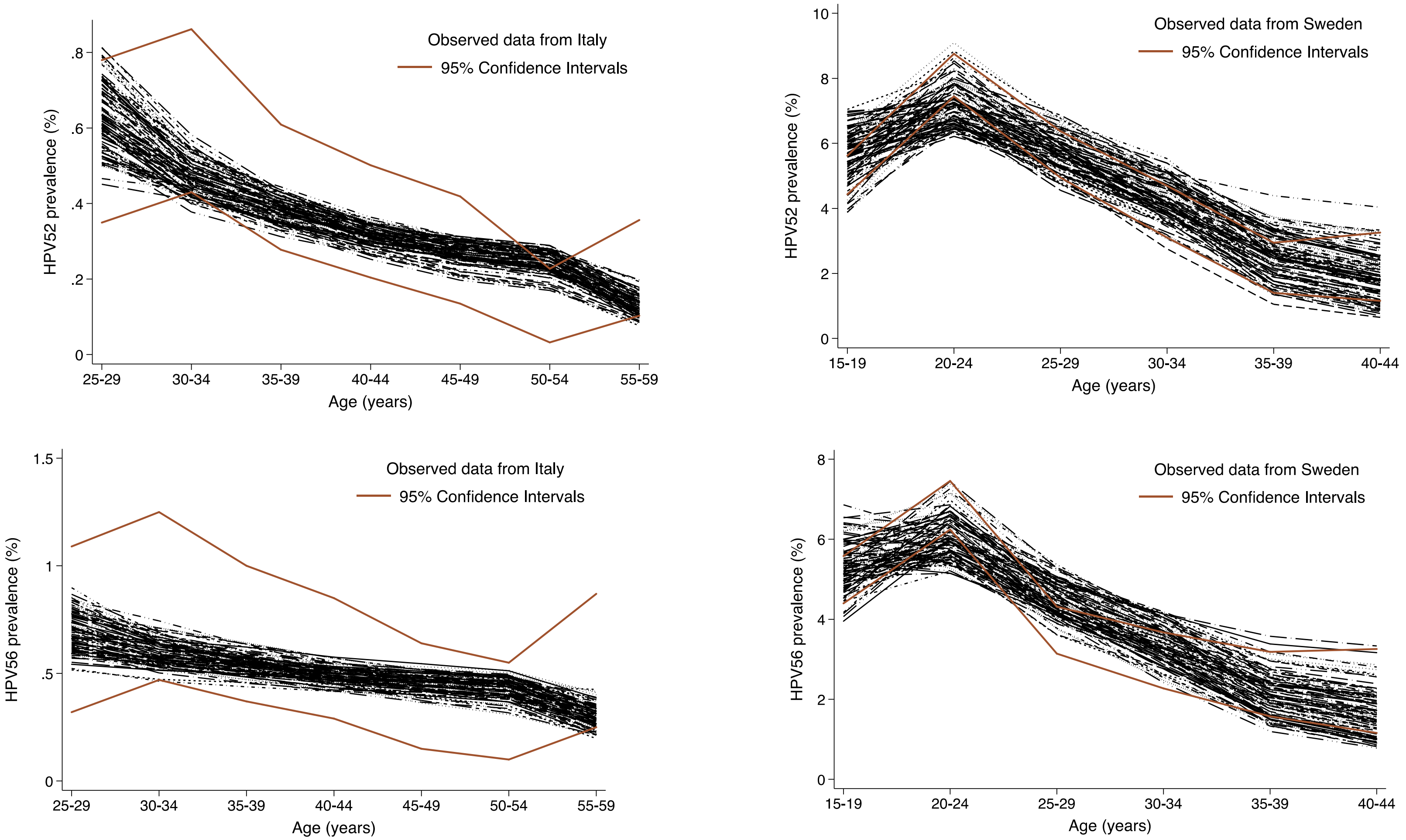

Figure S2.7. Fit between observed age-specific HPV58 and 59 prevalence and the 100 best fitting estimated curves, by country.

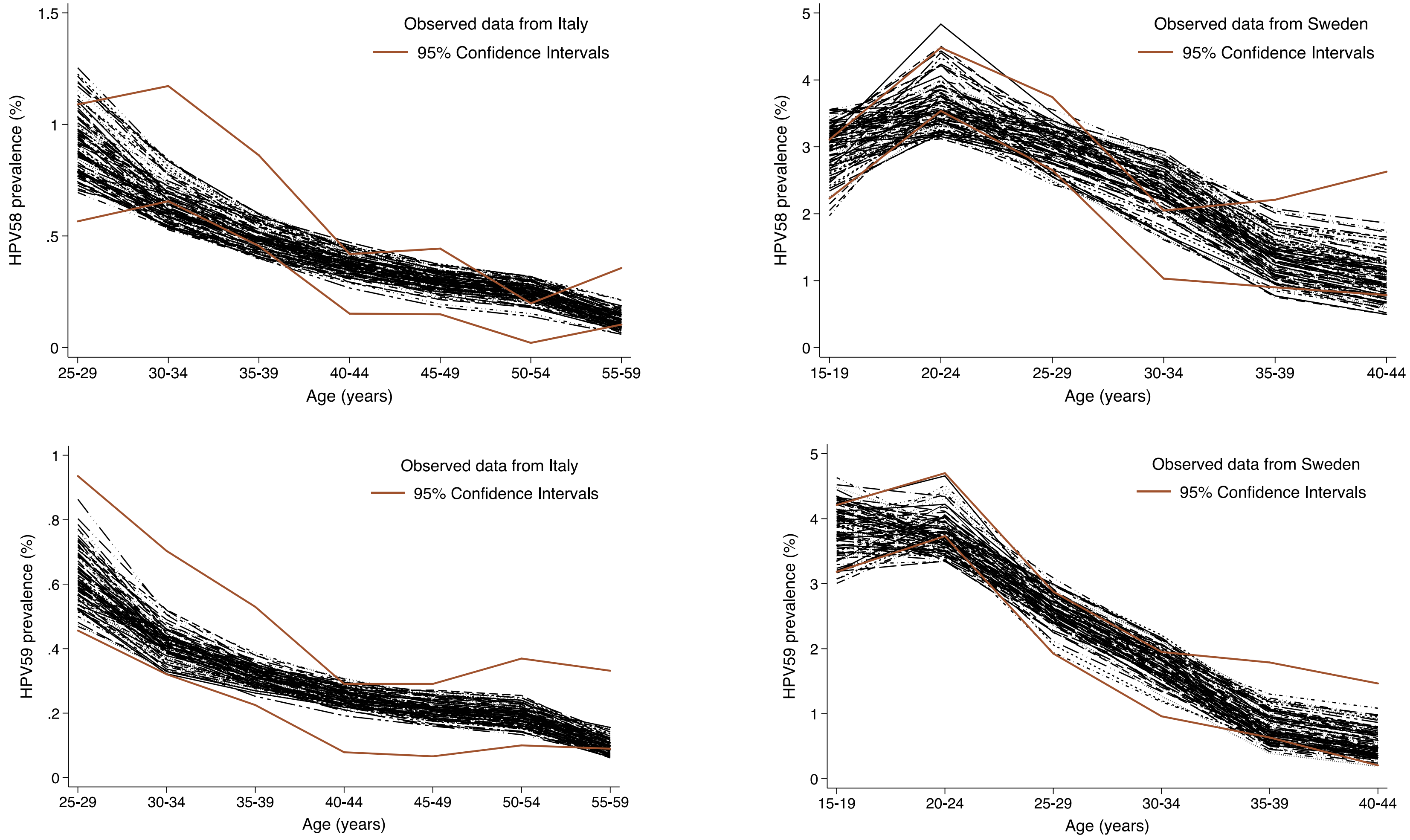

Figure S2.8. Fit between observed age-specific HPV68 prevalence and the 100 best fitting estimated curves, by country.

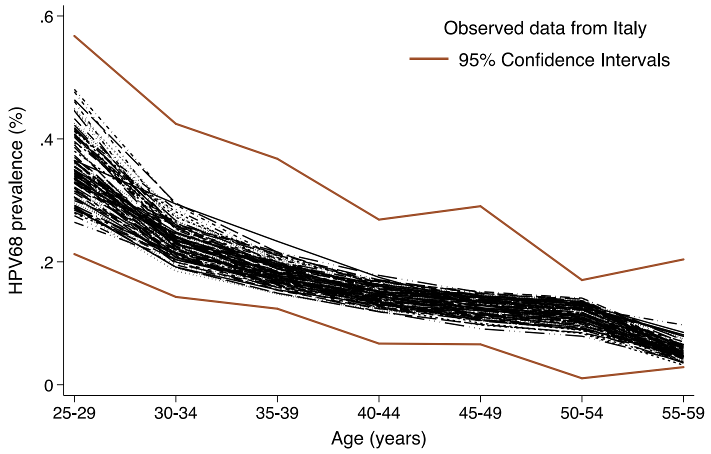


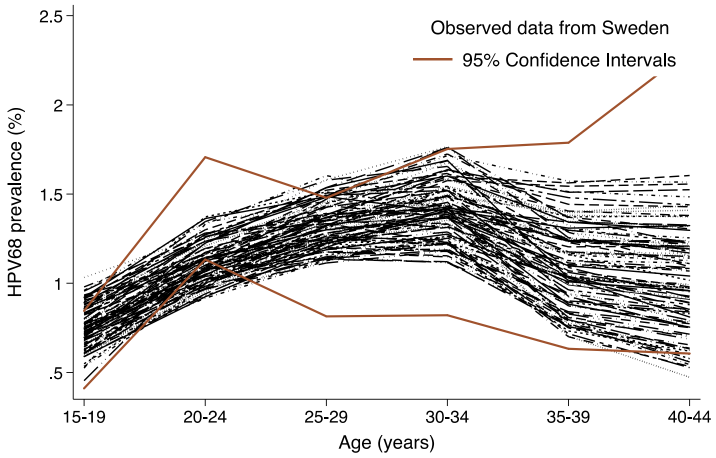

Supplement: File S6 — Figures S2.5-S2.8. Fit between prevalence curves and model outputs by country- Part B. (PDF) [file pone.0081171.s006.pdf]
