# Supplementary material for: Type-Specific Human Papillomavirus Biological Features: Validated Model-Based Estimates
Source: PLoS One. 2013 Nov 29;8(11):e81171. doi: 10.1371/journal.pone.0081171 (PMC3882251; doi:10.1371/journal.pone.0081171)
Supplement: File S7 — Figures S2.9-S2.14. Results of univariate and bivariate analyses. (PDF) [file pone.0081171.s007.pdf]

Figure S2.9: Sensitivity analysis for HPV16 model, Italy. Univariate quadratic regressions: dependent variable log-likelihood, independents variables input parameters.

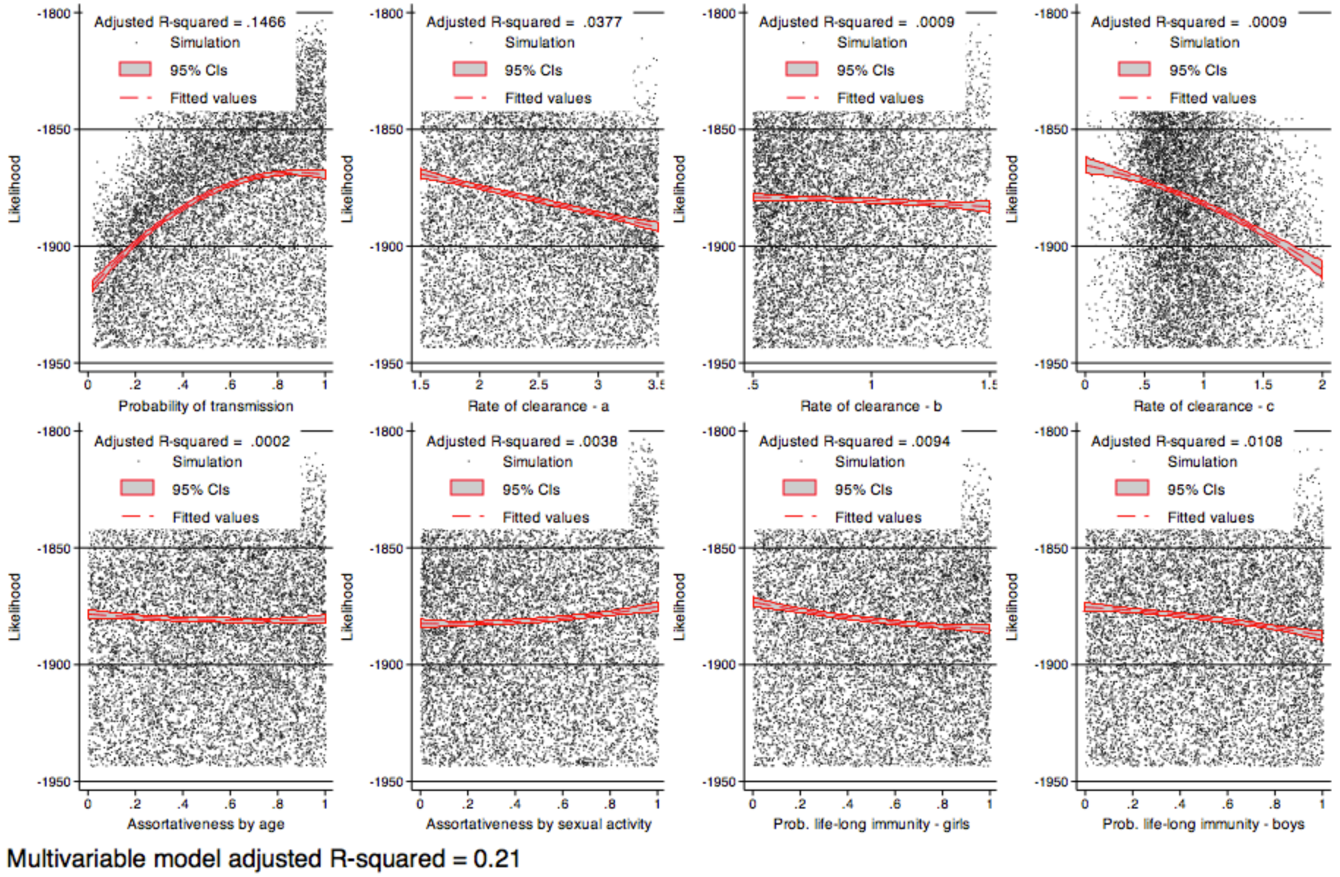

Figure S2.10: Sensitivity analysis for HPV16 model, Sweden. Univariate quadratic regressions: dependent variable log-likelihood, independents variables input parameters.

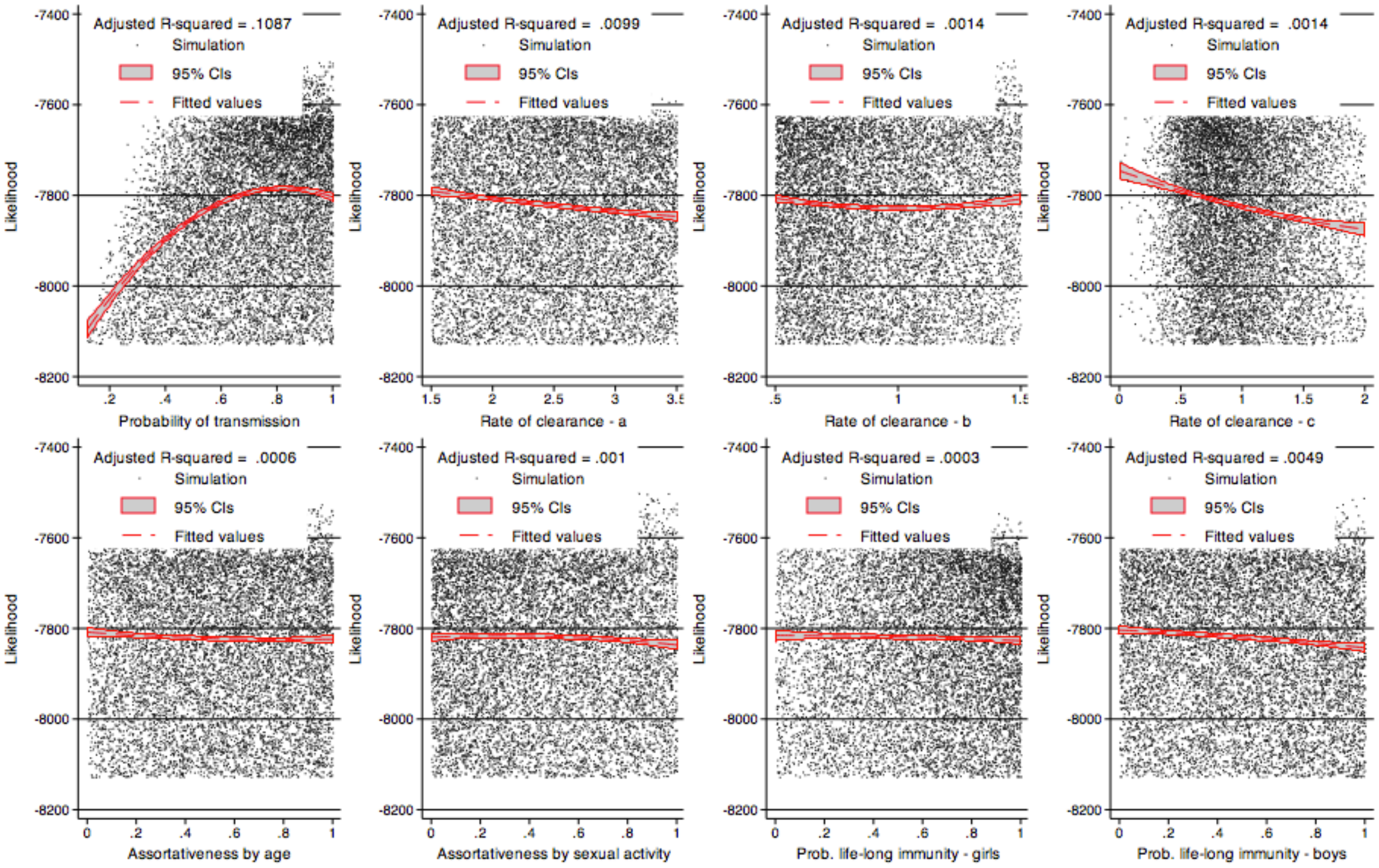

Multivariable model adjusted R-squared = 0.16

Figure S2.11: Sensitivity analysis for HPV18 model, Italy. Univariate quadratic regressions: dependent variable log-likelihood, independents variables input parameters.

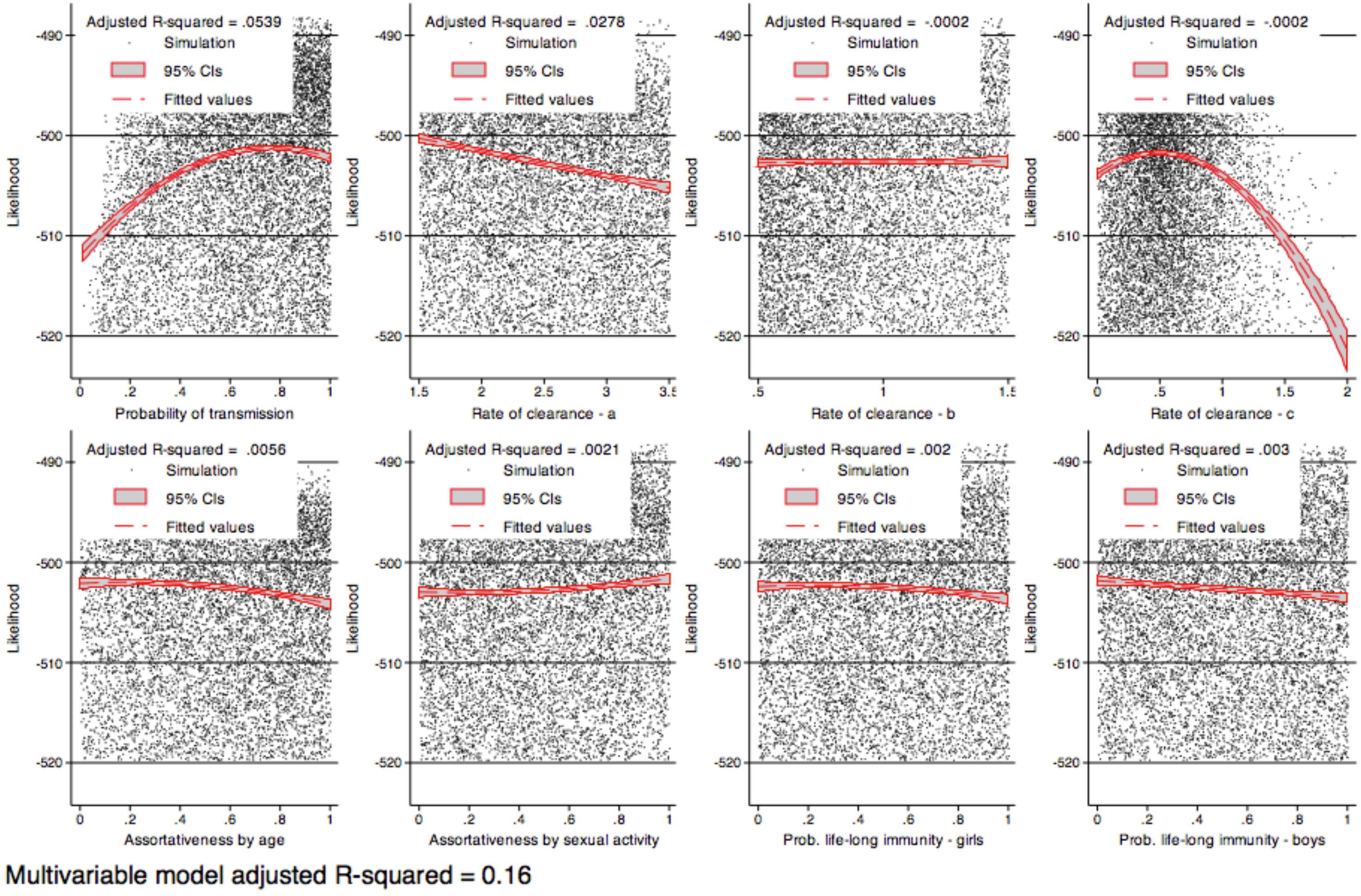

Figure S2.12: Sensitivity analysis for HPV18 model, Sweden. Univariate quadratic regressions: dependent variable log-likelihood, independents variables input parameters.

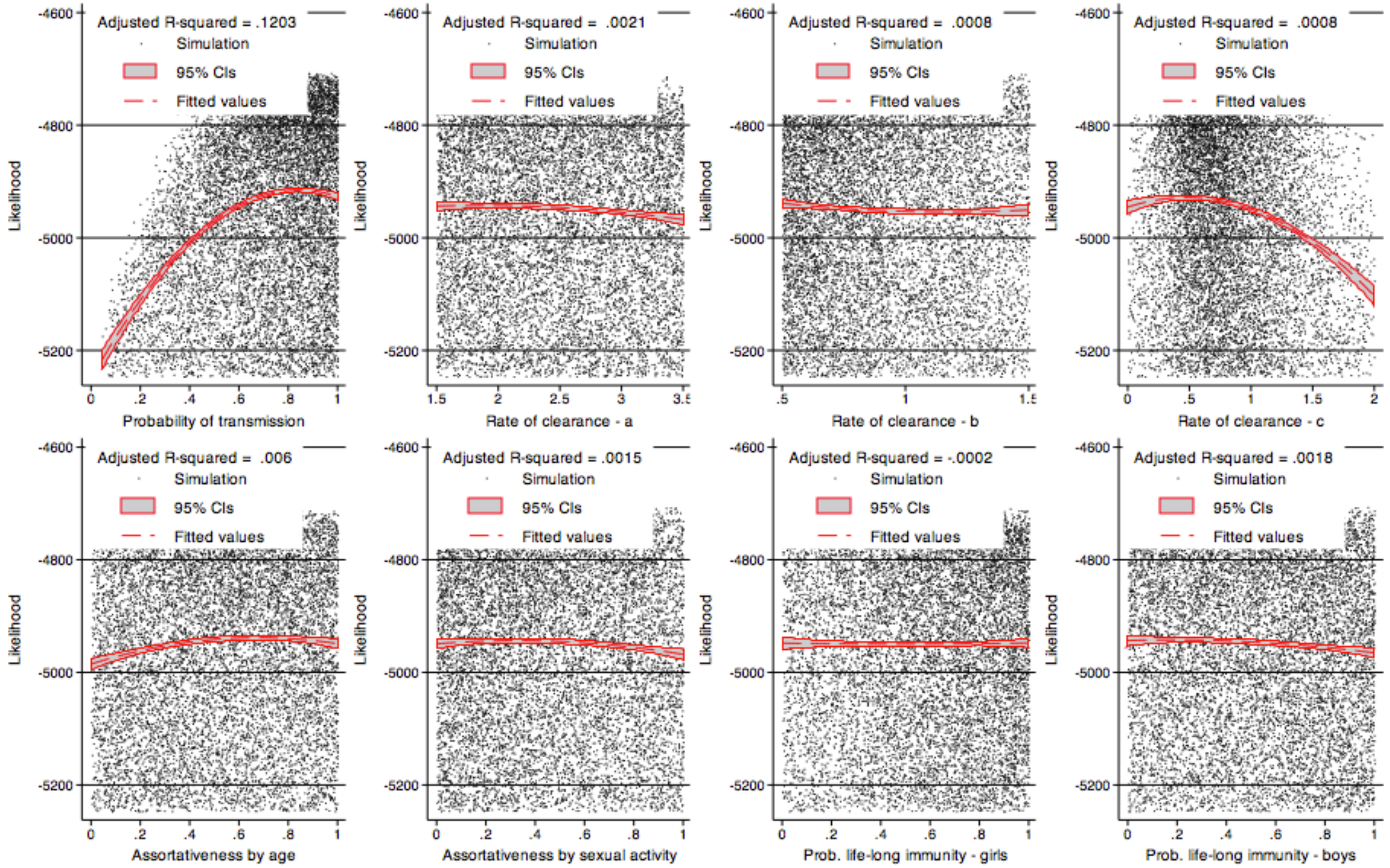

Multivariable model adjusted R-squared = 0.16

Figure S2.13: Sensitivity analysis for HPV16 model. Bivariate quadratic regressions: dependent variable log-likelihood, independents variables input parameters.

Italy

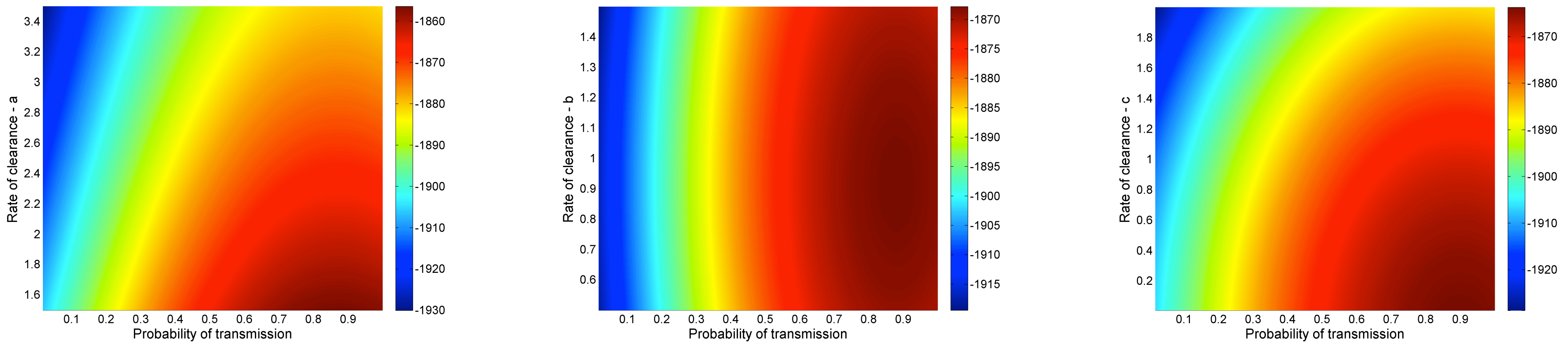

Adjusted R-squared: 0.188 Adjusted R-squared: 0.147 Adjusted R-squared: 0.161

Sweden

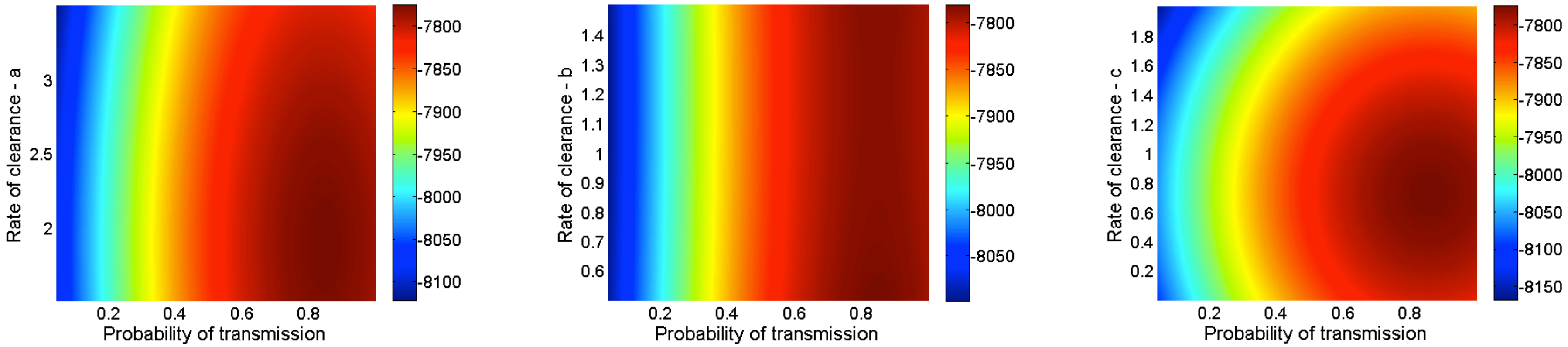

Adjusted R-squared: 0.124 Adjusted R-squared: 0.110 Adjusted R-squared: 0.118

Figure S2.14: Sensitivity analysis for HPV18 model. Bivariate quadratic regressions: dependent variable log-likelihood, independents variables input parameters.

Italy

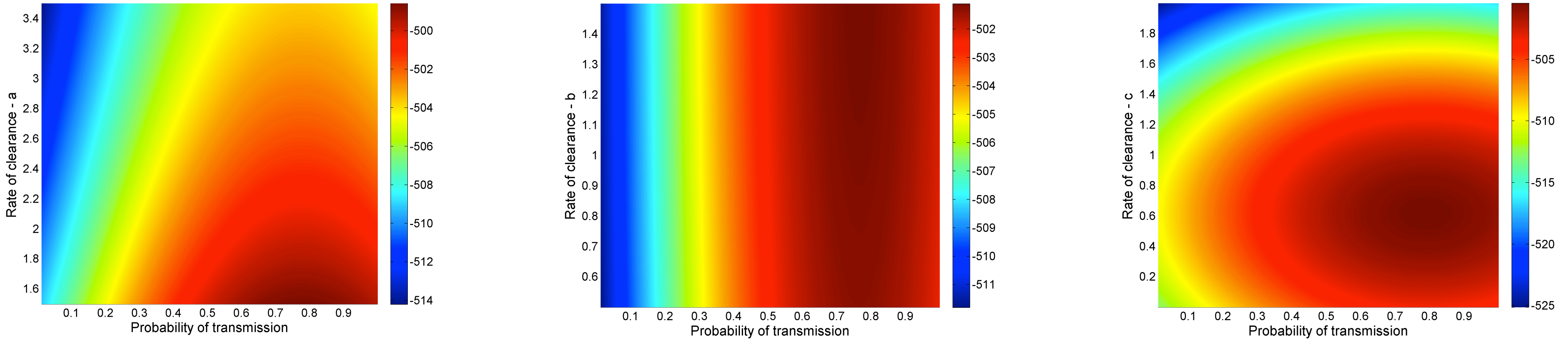

Adjusted R-squared: 0.084

Adjusted R-squared: 0.054

Adjusted R-squared: 0.073

Sweden

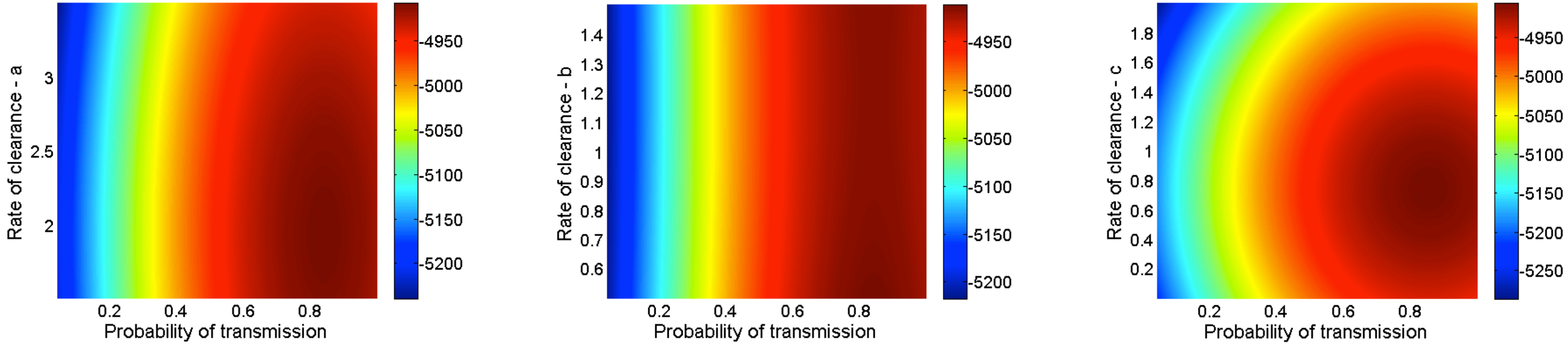

Adjusted R-squared: 0.123

Adjusted R-squared: 0.120

Adjusted R-squared: 0.131
