# Supplementary material for: Type-Specific Human Papillomavirus Biological Features: Validated Model-Based Estimates
Source: PLoS One. 2013 Nov 29;8(11):e81171. doi: 10.1371/journal.pone.0081171 (PMC3882251; doi:10.1371/journal.pone.0081171)

Figure S2.15. Age-specific HPV16, 18, 31, and 33 prevalence (%), by country.

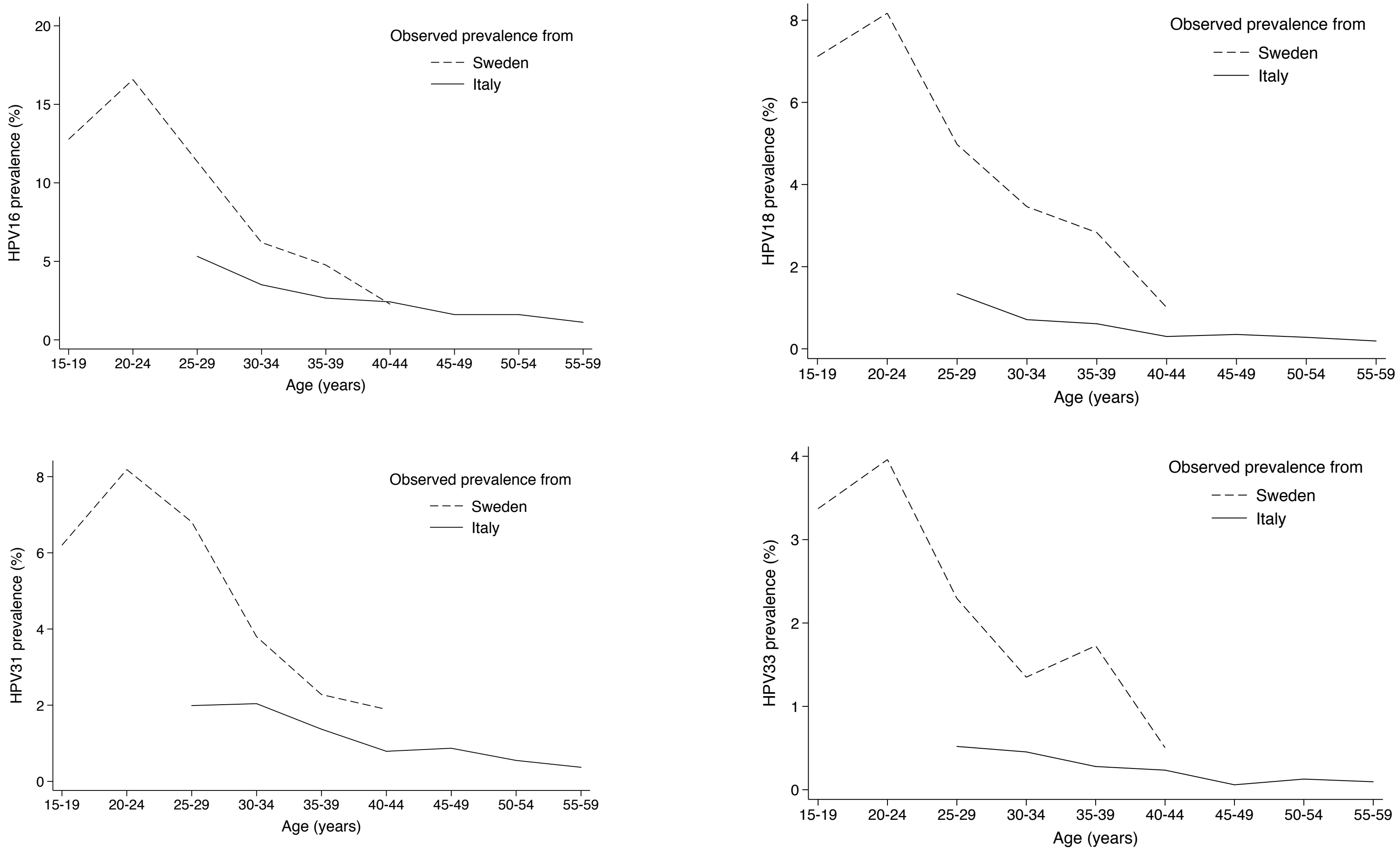

Figure S2.16. Age-specific HPV35, 39, 45, and 51 prevalence (%), by country.

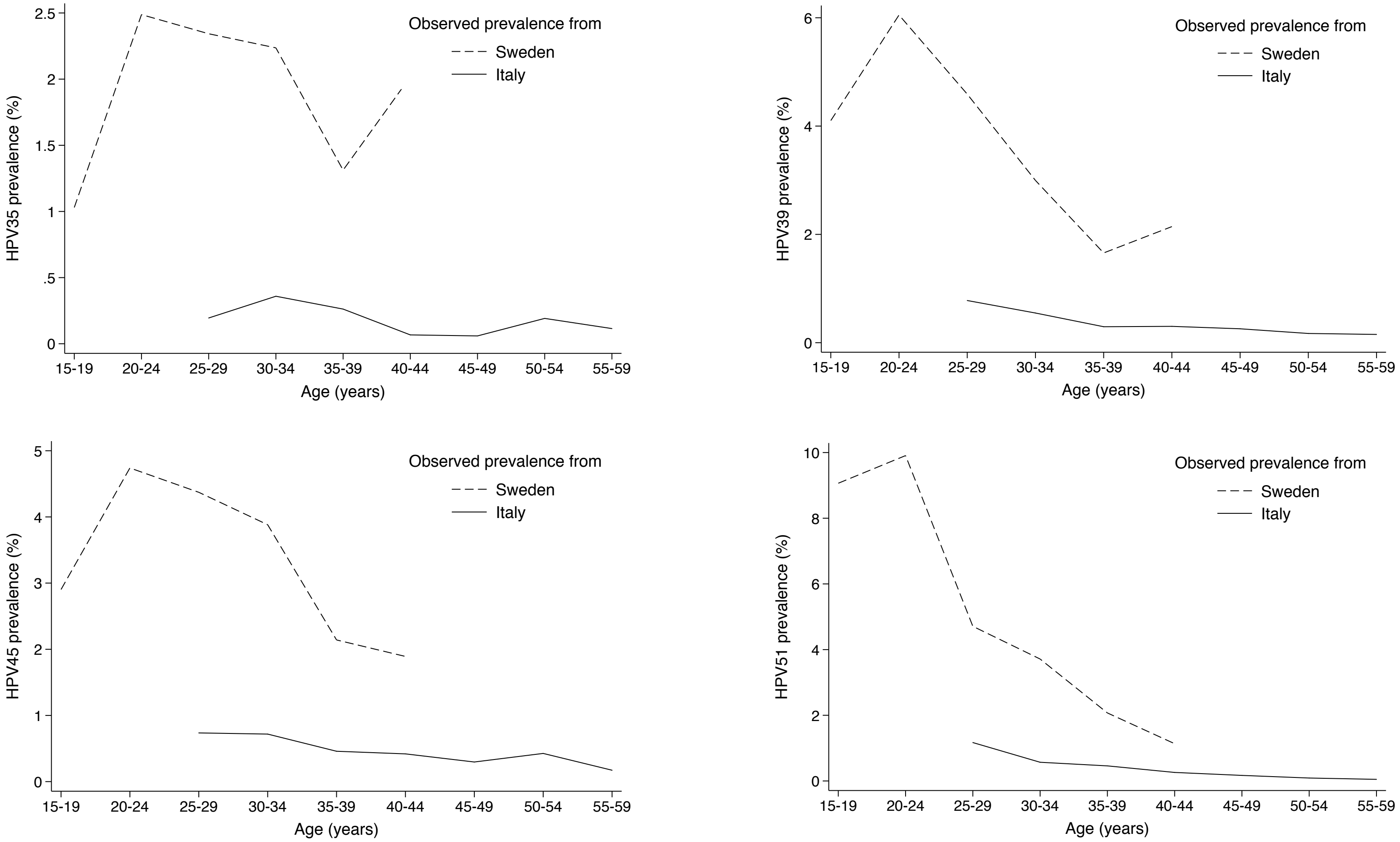

Figure S2.17. Age-specific HPV52, 56, 58, and 59 prevalence (%), by country.

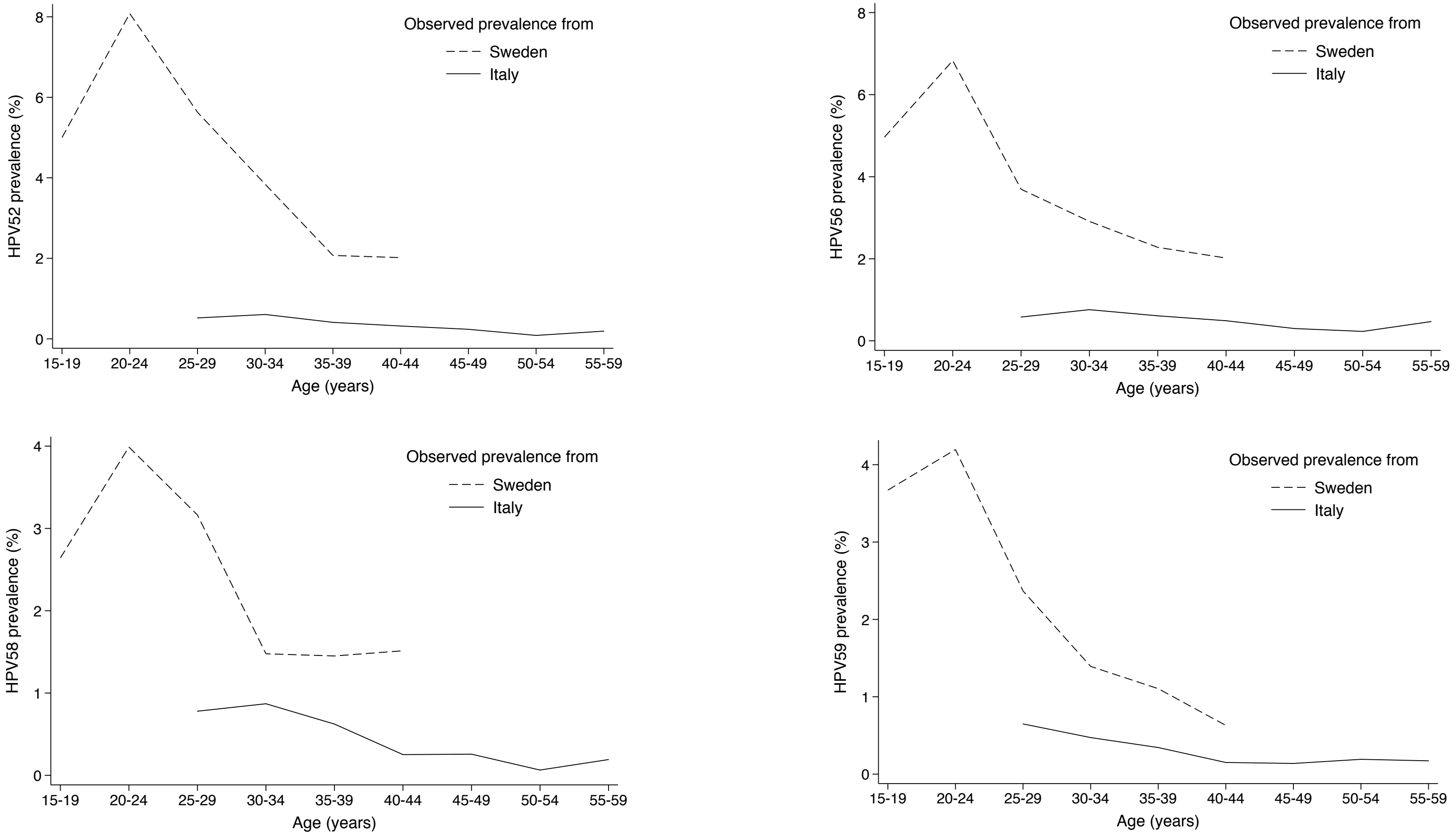

Figure S2.18. Age-specific HPV68 prevalence (%), by country.

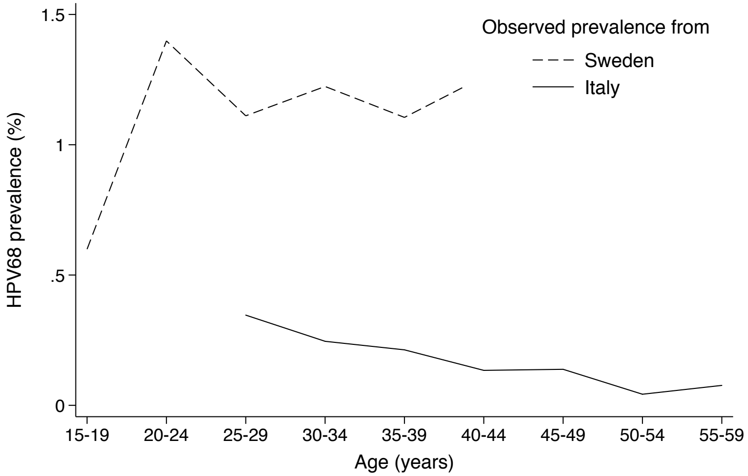

Supplement: File S8 — Figures S2.15-S2.18. Type-specific HPV prevalence curves by country. (PDF) [file pone.0081171.s008.pdf]
