# Supplementary material for: Type-Specific Human Papillomavirus Biological Features: Validated Model-Based Estimates
Source: PLoS One. 2013 Nov 29;8(11):e81171. doi: 10.1371/journal.pone.0081171 (PMC3882251; doi:10.1371/journal.pone.0081171)
Supplement: File S9 — Table S3.1. Summary of published models. (PDF) [file pone.0081171.s009.pdf]

Table S3.1. Summary of selected characteristics (HPV type investigated, probability of transmission per sexual partnership, rates and patterns of clearance, and method of finding validation) of published dynamic transmission models.

Publication	HPV type	Transmission per sexual partnership (or per intercourse*)		Rate of clearance (or rate of loss of natural immunity**)			Validation
		Values (probability)	Source	Pattern of immune response following infection clearance (%)	Values (person-year)	Source	
Hughes et al., 2002 (*12*)	HR	Males to females: 0.8 Females to males: 0.7	Assumed	SIR	0.67	Assumed	Not reported
Taira et al., 2004 (*13*)	16/18	Decreasing with age: 0.35 to 0.15	Calibrated	SIS	Decreasing with age: 0.67 to 0.07	Calibrated	Against published data
Barnabas et al., 2006 (*16*)	16	0.6	Calibrated	SIR	HPV infection 15-29 yrs: 0.4 30+ yrs: 0.1 LSIL clearance 15-34: 0.1 35+: 0.06	Assumed	Not reported

Publication	HPV type	Transmission per sexual partnership (or per intercourse*)		Rate of clearance (or rate of loss of natural immunity**)			Validation
		Values (probability)	Source	Pattern of immune response following infection clearance (%)	Values (person-year)	Source	
Kim et al., 2007 (*20*)	16	0.39	Calibrated	SIS/SIR (50.5/49.5)	0.9	Calibrated	Against published data
	18	0.33	Calibrated	SIS/SIR (53.3/46.7)			
Elbasha et al., 2007 (*14*)	16/18	Males to females: 0.8	Assumed	SIR	0.83	Assumed	Against published data
		Females to males: 0.7	Assumed				
Choi et al., 2010 (*42*)	16	0.36	Calibrated	SIS or SIR	Decreasing with time elapsed since infection: values not reported	Calibrated	Not reported
	18	0.18					
Bogaaards et al., 2010 (*15*)	16	0.8	Calibrated	SIRS	0.041**	Calibrated	Not reported
	18	0.93			0.029**		
	31	0.74			0.04**		
	33	0.9			0.037**		
	35	0.92			0.033**		
	39	0.71			0.027**		
	45	0.79			0.038**		
	51	0.9			0.037**		

Publication	HPV type	Transmission per sexual partnership (or per intercourse*)		Rate of clearance (or rate of loss of natural immunity**)			Validation
		Values (probability)	Source	Pattern of immune response following infection clearance (%)	Values (person-year)	Source	
	52	0.83			0.024**		
	56	0.9			0.047**		
	58	0.74			0.043**		
	59	0.85			0.016**		
	66	0.94			0.032**		
	68	0.43			0.014**		
Zechmeister et al., 2009 (*26*)	16<br>18<br>HR	0.6<br>0.1<br>0.3	Calibrated	SIRS	0.9<br>1.3<br>0.6	Assumed	Against published data
Olsen J et al., 2010 (*25*)	16<br>18	0.3*<br>0.13*	Assumed	SIS/SIR (55/45)	HPV to Sus: 0.92<br>CIN1 to Sus: 0.33<br>HPV to Sus: 1.1<br>CIN1 to Sus: 0.33	Assumed	Against published data
Van de Velde et al., 2010 (*21*)	16	Males to females: 0.88*<br>Females to males: 0.72*	Calibrated	Girls: SIS/SIR (54/46)<br>Boys: SIS/SIR (45/55)	Range (by age)<br>Girls: 0.6-1.7<br>Boys: 0.4-1.7	Calibrated	Against data not used for fitting

Publication	HPV type	Transmission per sexual partnership (or per intercourse*)		Rate of clearance (or rate of loss of natural immunity**)			Validation
		Values (probability)	Source	Pattern of immune response following infection clearance (%)	Values (person-year)	Source	
	18	Males to females: 0.47*<br>Females to males: 0.37*			Relative clearance rates: 1.25 (HPV16 reference category)		
	HR	Males to females: 0.36*<br>Females to males: 0.27*			Relative clearance rates: 1.17 (HPV16 reference category)		
Baussano et al., 2011 (*19*)	16	0.4<br>Decreasing with age: 0.28 to 0.11	Calibrated	SIR<br>SIS	Decreasing with time elapsed since infection: 1.6 to 0.036	Calibrated	Against published data
Johnson et al., 2012 (*22*)	16<br>18<br>31<br>33	0.72<br>0.74<br>0.74<br>0.75	Calibrated	SIRS	0.24**<br>0.17**<br>0.18**<br>0.18**	Calibrated	Not reported

Publication	HPV type	Transmission per sexual partnership (or per intercourse*)		Rate of clearance (or rate of loss of natural immunity**)			Validation
		Values (probability)	Source	Pattern of immune response following infection clearance (%)	Values (person-year)	Source	
	35	0.75			0.16**		
	39	0.75			0.21**		
	45	0.74			0.16**		
	51	0.75			0.19**		
	52	0.74			0.27**		
	56	0.74			0.17**		
	58	0.76			0.20**		
	59	0.74			0.15**		
	66	0.76			0.17**		
Vanni et al., 2012 (*41*)	16<br>18	0.55<br>0.52	Calibrated	SIS	0.52	Calibrated	Not reported

HR: High-risk; SIS: susceptible-infected-susceptible; SIR susceptible-infected-recovered/immune; SIRS: susceptible-infected-recovered/immune-susceptible
